# Integrative gene network analysis identifies key signatures, intrinsic networks and host factors for influenza virus A infections

**DOI:** 10.1038/s41540-017-0036-x

**Published:** 2017-12-04

**Authors:** Christian V. Forst, Bin Zhou, Minghui Wang, Tsui-Wen Chou, Guy Mason, Won-min Song, Eric Schadt, Elodie Ghedin, Bin Zhang

**Affiliations:** 1 Department of Genetics and Genomic Sciences, Icahn Institute of Genomics and Multiscale Biology, Icahn School of Medicine at Mount Sinai, 1470 Madison Avenue, New York, NY 10029 USA; 2 Center for Genomics & Systems Biology, Department of Biology, New York University, 12 Waverly Place, New York, NY 10003 USA; 3 College of Global Public Health, New York University, 12 Waverly Place, New York, NY 10003 USA

## Abstract

Influenza A virus, with the limited coding capacity of 10–14 proteins, requires the host cellular machinery for many aspects of its life cycle. Knowledge of these host cell requirements not only reveals molecular pathways exploited by the virus or triggered by the immune system, but also provides further targets for antiviral drug development. To uncover novel pathways and key targets of influenza infection, we assembled a large amount of data from 12 cell-based gene-expression studies of influenza infection for an integrative network analysis. We systematically identified differentially expressed genes and gene co-expression networks induced by influenza infection. We revealed the dedicator of cytokinesis 5 (*DOCK5*) played potentially an important role for influenza virus replication. CRISPR/Cas9 knockout of *DOCK5* reduced influenza virus replication, indicating that *DOCK5* is a key regulator for the viral life cycle. *DOCK5*’s targets determined by the *DOCK5* knockout experiments strongly validated the predicted gene signatures and networks. This study systematically uncovered and validated fundamental patterns of molecular responses, intrinsic structures of gene co-regulation, and novel key targets in influenza virus infection.

## Virology: Molecular Networks and Targets in Influenza Infection

Molecular response to influenza infection involves a large number of interacting pathways in the form of complex molecular networks. Most studies on influenza infection have largely focused on testing specific molecules and hypotheses with limited data. Therefore, a global picture of molecular interactions in influenza infection is missing. In this study, we performed an integrative network analysis on a large amount of data from 12 cell-based gene expression studies of influenza infections. By combining differential expression, co-expression networks, and gene knockout experiments, we uncovered and validated fundamental patterns of molecular responses, intrinsic structures of gene co-regulation, and novel key targets in influenza infection. Our findings pave the way for other functional investigations into identifying novel therapeutic targets against influenza infection.

## Introduction

The influenza A virus (IAV), a member of the *Orthomyxoviridae* family, is the causal agent of an acute respiratory tract infection suffered annually by 5–20% of the human population. IAV can cause high mortality in humans, with 250,000–500,000 deaths per year worldwide.^1^ In pandemic years, influenza infection can lead to even higher mortality rates, as seen in the most extreme case with the 1918 Spanish influenza pandemic.^2^ Of particular concern is the threat of emerging highly pathogenic avian influenza viruses such as H5N1 and H7N9, which—although not easily transmissible human-to-human—have an unusually high death rate. Current treatments are focused on vaccines and drugs that target viral proteins. However, both of these approaches have limitations as vaccines require annual development to match the antigenic strains circulating, while viral proteins have an impressive capacity to evolve resistance against anti-viral agents.^3^ With the expression of 14 functional proteins for viral replication and virulence, the repertoire of gene products on the pathogen side is limited. The viral life cycle and the replication of the IAV are dependent on hijacking host-cell biological processes to facilitate entry, replication, assembly, and budding. The recognition that a suite of mammalian host proteins is required for IAV infection and replication presents additional targeting strategies that may be less prone to deflections by the quickly mutating viral genome.

IAV entry is a dynamic process that is comprised of six different steps:^4^ (i) attachment to the target cell, (ii) internalization into cellular compartments, (iii) endosomal trafficking to the perinuclear region, (iv) fusion of viral and endosomal membranes, (v) uncoating, and (vi) import of the viral genome into the nucleus. After nuclear import three more steps are required: genome replication/transcription and translation; vRNP transport from the nucleus to the cytoplasm; and virus assembly and release.^5^

Influenza infection activates a number of host defense pathways, including the innate and adaptive immune responses, the induction of cytokines, and activation of apoptosis.^6^ The detection of viral particles (in particular nuclear acids) by toll-like receptors (*TLR7*) of the *MyD88*, *NF-kB* pathway,^7,8^ as well as cytosolic proteins such as *RIG-I* (*DDX58*)^9,10^ of the *MDAF*/*MAVS* pathway and their trigger of interferon expression via the activation of transcription factors including *IRF3* and *IRF7*, have been well studied. Unfortunately, much less is known about downstream host defense factors and signaling pathways.

Large-scale genome-wide studies of viral host factors and corresponding cellular networks have been conducted since 2008. Because RNAi-based screening technology was not well established in mammalian cells at that time, *Drosophila* was tested as an experimental platform to characterize host–virus interactions during influenza infections.^11^ With the development of mammalian RNAi-based screening, a comprehensive analysis of mammalian host cell functions in influenza virus replication became feasible.^12–14^ Known protein–protein interactions were used and superimposed with the RNAi screening data to construct functional host–pathogen interaction networks relevant for the influenza life cycle. A different tactic was then employed^15^ to combine yeast two-hybrid analyses, genome-wide transcriptional gene expression profiling, and an RNAi screening. More recent approaches revisited transcriptomic data but used weighted gene co-expression network analysis (WGCNA) to construct a host-influenza regulatory network.^16^

In this study, we employed an integrative network-based approach to identify host response co-expression networks in influenza A virus infection. Integration of differential gene expression, data driven correlation, and co-expression networks enabled reconstruction of novel signaling maps underlying influenza infection and host response. We predicted and validated *DOCK5* as a key driver and potential host factor that is important for influenza virus infection.

## Results

Twelve cell-based time-series gene expression data sets from ten published studies were assembled (Table 1) to identify key processes and key regulators in influenza infections. Cell lines, such as human alveolar basal epithelial cells (A549) and cultured human airway epithelial cells (Calu-3), as well as primary human bronchial epithelial cells (HBEC) were used to capture the overall response of the primary target tissues after influenza infection. The influenza A viruses used for infection include strains of subtypes H1N1 (including the resurrected strain of the 1918 pandemic), H5N1, H5N2, and H9N2. The cell-based experimental platforms include time-series data up to 72 h post-infection but the majority cover time post-infection to 24 h. Thus this study focused primarily on the initial steps of the influenza infection and the host innate immune response.

**Table 1:** Human cell-based gene expression profiles obtained from the GEO database

Expression set	Model	Virus	MOI^a^	# Probes	# Sig. hits	%
GSE19392	HBEC	PR8 (H1N1)	5	22,277	546	2.5
GSE28166	Calu-3	H5N1	1	41,000	1704	4.2
GSE31524	A549	A/WSN/33 (H1N1)	4	54,675	408	0.8
	subsets	A/duck/Malaysia/F118/08/2004 (H5N2)			265	0.5
		A/duck/Malaysia/01 (H9N2)			130	0.2
GSE33142	Calu-3	A/VN/1203/04 (H5N1)	1	45,015	401	0.9
GSE36555	A549	A/Mex/inDRE4487/2009 (H1N1)	0.1	49,707	1756	3.5
GSE37571	Calu-3	A/CA/04/2009 (H1N1)	3	41,000	2144	5.2
GSE37951	A549	r1918, NS1 Tx/91 (H1N1)	2	41,000	11,679	28.5
	A549	r1918 (H1N1)	2	41,000	16,830	41.1
GSE40844	Calu-3	A/CA/04/2009 (H1N1)	3	41,000	576	1.4
	Calu-3	A/Neth/602/2009 (H1N1)	3	41,000	1875	4.6

^a^ Multiplicity of infection

**Fig. 1:**
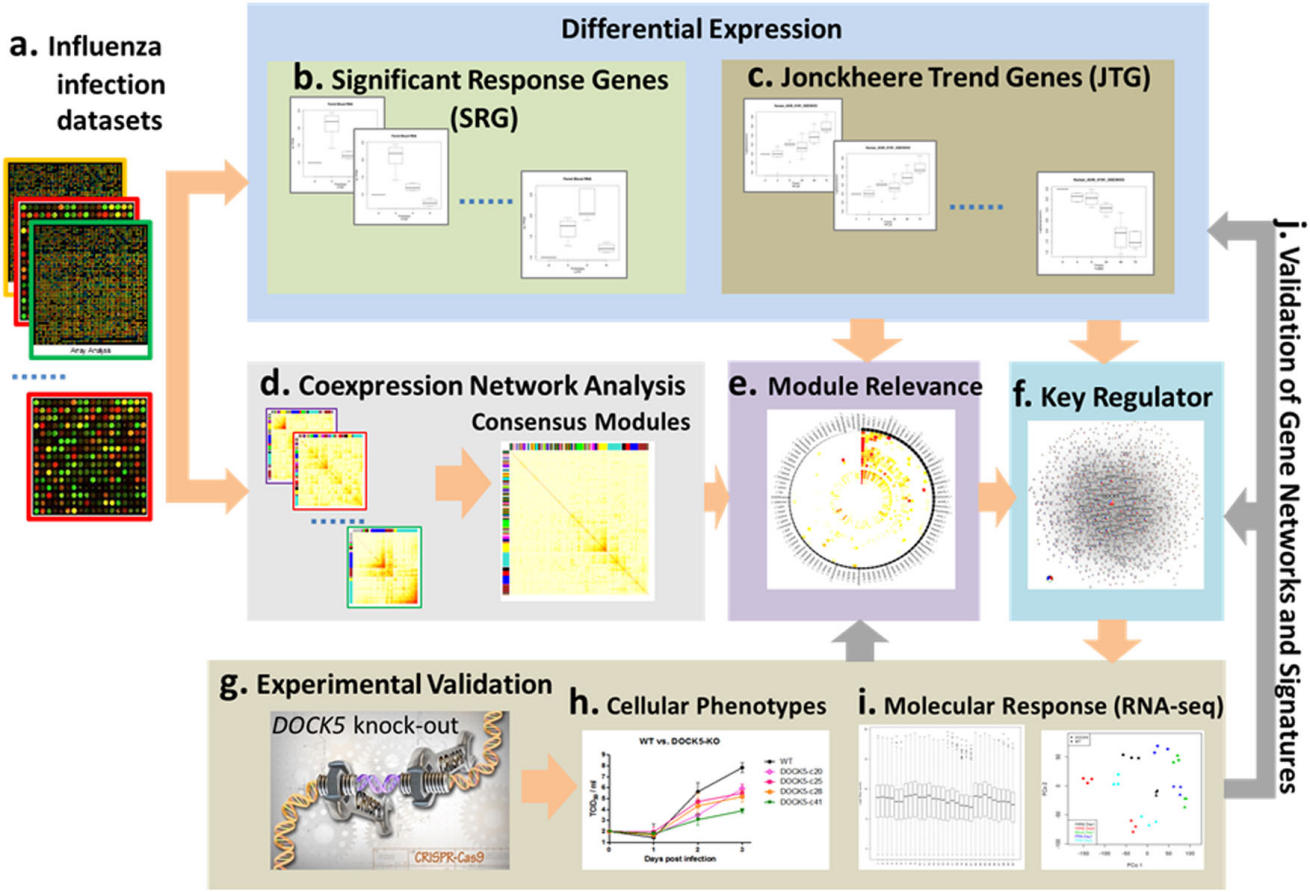
Overview of the proposed integrative network-based approach to influenza infection. **a** Twelve publically available gene expression data sets for studying influenza infection were curated. **b** ANOVA was used to identify significant response genes (SRG) that were differentially expressed by taking into account the time-series. **c** Genes showing significant up-regulation or down-regulation along the time-series were identified by the Jonckheere trend analysis and they were called Jonckheere trend genes (JTG). **d** Weighted co-expression network analysis was applied to each data set to identify modules comprised of highly co-regulated genes. Consensus modules were further determined by the clustering analysis of the similarities between all the modules from the 12 data sets. **e** The consensus modules were rank-ordered by their enrichment for both SRGs and JTGs. **f** Key regulators of the top-ranked module were determined by network connectivity. Key driver centered un-weighted co-expression networks were then constructed by correlation analysis of the individual data sets. **g** Key regulator (*DOCK5*) predicted by the network analysis was validated through CRISPR-Cas9-based knockout experiments. **h** The impact of *DOCK5* knockout on the replication of the virus in A549 cells was determined by titration of the culture supernatants using a TCID50 assay. **i** Molecular response to *DOCK5* knockout, i.e., *DOCK5*’s target genes, was determined by RNA sequencing data from the CRISPR-Cas9-based knockout experiments. **j** The predicted gene networks and differentially expressed gene signatures were validated by their enrichment of *DOCK5* target genes identified through the validation experiments

These data sets were processed separately by an integrative network analysis approach (Fig. 1), which was primarily based on weighted gene co-expression networks.^17,18^ We first identified differentially expressed genes in each data set that showed significant change (significant response genes, SRG) or trend (Jonckheere Trend Genes, JTG) during influenza infection. Individual sets of differentially expressed genes (DEG) were further assembled to consensus sets. Weighted co-expression network analysis was applied to each data set to identify modules of highly co-regulated genes that were further analyzed to derive consensus modules through a clustering analysis of the module–module similarity matrix (see details in the Methods section). The consensus modules were prioritized by their enrichment for both SRGs and JTGs, and the key regulators (hub genes) in each module were determined by network connectivity. A top key regulator (*DOCK5*) of the top ranked module involved in viral replication was validated through CRISPR-Cas9-based knockout experiments. The impact of *DOCK5* knockout on the replication of the virus in A549 cells was determined by titration of the culture supernatants using a TCID50 assay. Molecular responses to *DOCK5* knockout, i.e., *DOCK5*’s target genes, were determined by RNA sequencing of the *DOCK5-*knockout cell lines and were used to validate the predicted gene networks and differentially expressed gene signatures.

Gene set enrichment analysis with well-established gene sets from gene ontology (GO)^19^ and MSigDB^20^ were used to assess biological functions. Influenza-specific processes were evaluated by enrichment calculations using published gene sets including the influenza host factors,^5,21^ the inflammasome,^22^ the interferon stimulated genes (ISGs)^23^, and the known host defense factors from InnateDB.^24^ All *p* values reported in the text are corrected for multiple testing unless otherwise specified. These gene sets, along with a number of abbreviations used throughout the manuscript, are described in Table 2.

**Table 2:** Abbreviations used in this manuscript

Data set	Size	Description
SRG	Various	Differentially expressed gene identified by ANOVA time series analysis. SRGs(*n*) refers to the DEGs conserved in at least *n* data sets, *n* = 1,2,…,12
JTG	Various	Gene showing significant up- or down-trend across the time series identified by Jonckheere trend analysis. JTGs(*n*) refers to the genes with up-trend or down-trend in at least *n* data sets, *n* = 1,2,…,12
sgDOCK5-DEGS	Various	Gene signatures differentially expressed during influenza infection scenarios between *DOCK5*-wt and *DOCK5*-ko experiments (fold change ≥ 1.2 or ≤ 1/1.2 FDR < 0.05)
sgDOCK5-DEG^+^	2863	A gene signature up-regulated by *DOCK5* knockout (i.e., genes repressed by *DOCK5*) after H3N2 infection (absolute values used, fold change ≥ 1.2, FDR < 0.05)
sgDOCK5-DEG^-^	4512	A gene signature down-regulated by *DOCK5* knockout (i.e., genes activated by *DOCK5*) after H3N2 infection (absolute values used, fold change ≤ 1/1.2, FDR < 0.05)
DOCK5-CCGS(n)	Table S12	Genes correlated with *DOCK5* in at least *n* data sets, *n* = 2, 3,…, 12
GO	4653	Gene ontology of biological processes (BP)^19^
MSigDB	1329	Molecular signatures database, curated, canonical processes (c2.cp)^20^
Watanabe	129	Host-factors identified after consensus siRNA studies^5^
Ward	280	Targets after siRNA studies by Ward et al.^21^
Inflammatome	2483	Inflammatome gene set^22^
ISG	395	Interferon Stimulated Genes from the Interferome database^23^
InnateDB	1371	Genes from InnateDB^24^

### Differential expression analysis uncovers genes responding to influenza infection

Given the time series information across the different data sets, we were particularly interested in the temporal response of expressed genes. For this purpose, ANOVA was used to identify differentially expressed genes across the time series (SRGs; see Supplementary Information for details). In addition, a complementary non-parametric Jonckheere trend analysis^25^ was used to identify significant up-regulated or down-regulated genes across all measured time points (JTGs) using a threshold of 0.05 for the corrected *p*-values.

The conservation of SRGs and JTGs across the 12 data sets was then evaluated. Although the data sets include a variety of different cell lines infected with different influenza virus subtypes and strains, the resulting SRG signatures significantly overlap with each other based on the Fisher’s exact test (FET) (*p*-values range between 1.37e−11 and 0). As shown in Fig. S1, there are three groups without explicit preference of virus strain or cells, including (i) a large group of H1N1, H5N1, and H5N2 virus strains infecting A549 and Calu-3 cells; (ii) the H1N1/1918 strain with the original (gn37_8) and modified NS1 protein (gn37_7); and (iii) H9N2 avian influenza in A549 cells (gn31H9), and H1N1/PR8 in HBEC (gn19).

**Fig. 2:**
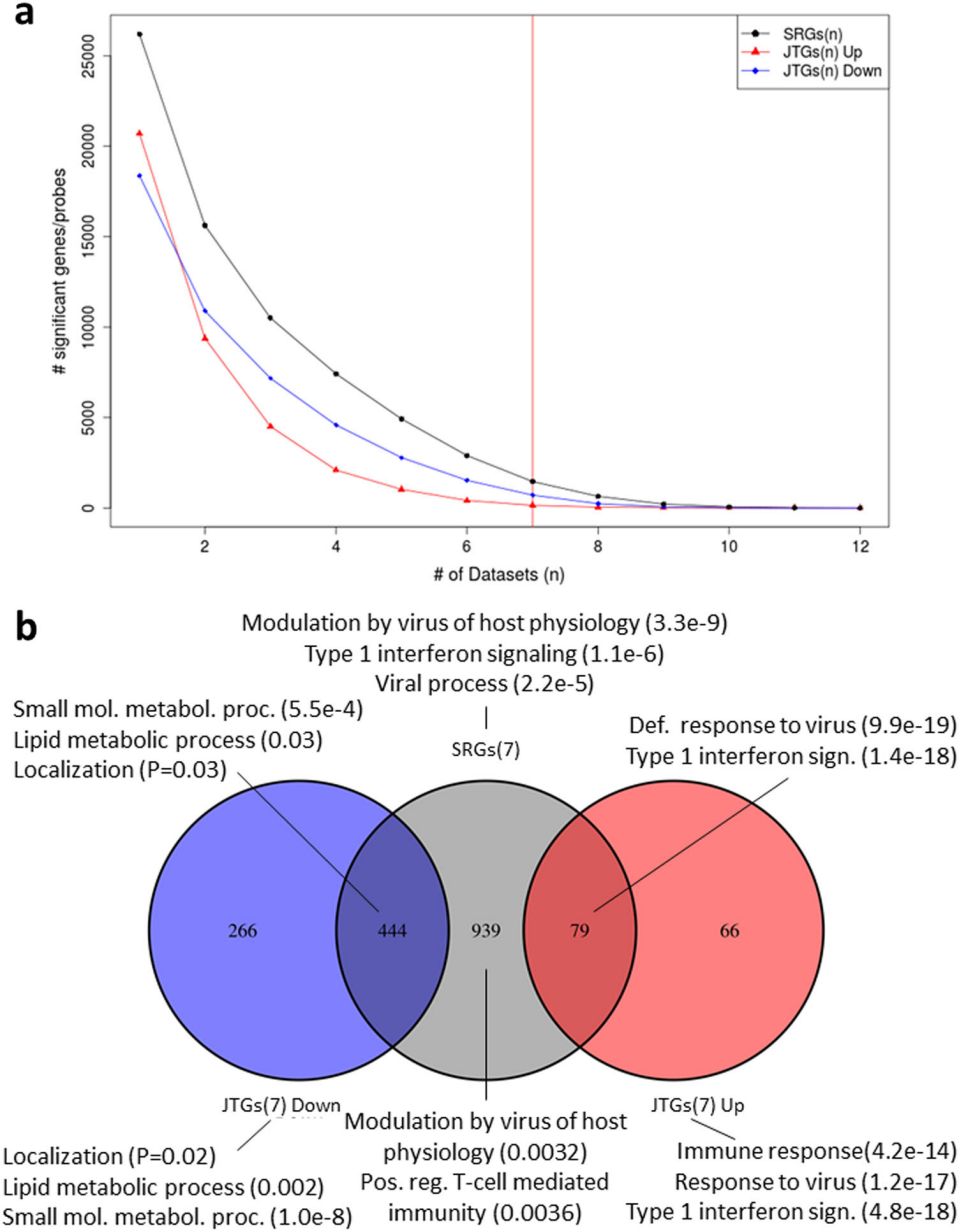
Conservation of the differentially expressed gene sets including ANOVA-based SRGs and Jonckheere trend analysis-based JTGs, across multiple data sets. SRGs(*n*) and JTGs(*n*) refer to the SRGs and JTGs shared by *n* data sets (*n* = 1, 2, …, *n*), respectively. **a** The number of SRGs(*n*) (black line), the number of up-regulated JTGs(*n*) (red line), and the number of down-regulated JTGs(*n*) (blue line). **b** A Venn diagram of the overlap between SRGs(7), and the up-regulated and down-regulated JTGs(7). Biological functions refer to the pathways enriched in the corresponding sets and the numbers in parentheses indicate the corrected FET *p*-values

We identified 2898 SRGs, including 416 up-regulated JTGs (JTGs^up^), 1526 down-regulated JTGs (JTGs^down^), and 956 non-JTGs, in at least 6 of the 12 studies at a false discovery rate of 5%, while 1462 SRGs (146 JTGs^up^, 711 JTGs^down^, and 605 non-JTGs) were common to at least seven studies (Fig. 2a). Enriched pathways were characterized by gene otology (GO) categories and MSigDB gene signatures (Fig. 2b; Table S1). As expected, the up-regulated genes are involved in immune system response, in particular type 1 interferon signaling (FET *p* = 1.09e−6, 3.9-fold). Well conserved are the host defense pathways comprised of *MX1*, *IFITM*, *IRF7*, and *OAS*, which are known ISGs that control IAV infections,^26^ among other interferon-induced genes. The down-regulated genes are associated with small molecule/lipid metabolic processes and localization. The heterogeneous nuclear ribonucleoprotein A1 (*hnRNPA1*) is the highest ranked gene significantly expressed in all 12 data sets and predominantly down-regulated (in 9/12 data sets). It is a member of a family of ubiquitously expressed hnRNPs, which are RNA-binding proteins associated with pre-mRNAs in the nucleus and that influence pre-mRNA processing, as well as other aspects of mRNA metabolism and transport. *HnRNPA1* is one of the most abundant core proteins of hnRNP complexes; it plays a key role in the regulation of alternative splicing.^27^ Together with splicing factor 2 (*SF2*), it regulates alternative splicing of interferon regulator factor-3 (*IRF3*).^28^ Mediated by transportin 1 (*TNPO1*),^29^ *hnRNPA1* shuttles between the nucleus and the cytosol.^30^ Other highly ranked members of the hnRNP family involve the related *hnRNPD* and the synaptotagmin binding cytoplasmic RNA interacting protein (*SYNCRIP*), both significantly expressed in 11 data sets. Although *SYNCRIP* is not known to be involved in influenza infection, it is a host factor involved in hepatitis C virus RNA replication,^31^ and required by the HCV IRES for translation-competent 48S complex formation.^32^ *SYNCRIP* has previously been reported to be associated with immune functions.^33^ Other SRGs, potentially responsible for host defense, include the *CD59* molecule (a cell surface glycoprotein that is involved in lymphocyte signal transduction), C–C motif chemokine ligand 5 (*CCL5*) and *MALT* paracaspase that may play a role in *NFκB* activation. A list of the best DEGs together with their consensus trend are shown in Table S2. Due to the diverse cell lines and virus strains used, even well conserved SRGs such as *hnRNPA1*, which is an SRG in all 12 data sets, show diverse gene expression with up-regulation in 2 and down-regulation in 9 data sets. Similarly, host defense genes experience diverse regulation depending on infected tissue and viral strain. For example, *IRF7* is up-regulated in 10/12 data sets, *MX1* is up-regulated in 9/12 data sets, and *RIG-I* (*DDX58*) is up-regulated in 7/12 and down-regulated in 3/12 data sets.

### Co-expression network analysis reveals intrinsic gene–gene co-regulation structures underlying influenza infection

To understand how the genes interact with each other during influenza infection, we performed WGCNA of the 12 data sets.^17,34,35^ The 12 weighted co-expression networks consist of a total of 1191 modules with 9–8529 members (Fig. S2). We further identified 282 consensus modules (CMs) of sizes between 20 and 2500 through an average link-based hierarchical clustering method using the Jaccard similarity measure for modules (see Supplemental Information). These consensus modules were ranked by the significance of the enrichment for the previously identified DEG signatures (see Methods and Table 3). The most important CM (turquoise) captures common system responses against influenza infections enriched for GO biological processes, such as translational elongation/termination (FET *p* = 4.7e−5, 4.8-fold) and viral reproduction (FET *p* = 4.0e−7, 2.3-fold). Enrichment analysis using the canonical pathway collection from MSigDB^20^ identifies similar virus response-related pathways, such as peptide chain elongation (FET *p* = 3.6e−5, 4.4-fold) and influenza life cycle (FET *p* = 2.5e−5, 3.6-fold) enriched in the top ranked consensus module. This turquoise CM is also most enriched for the known influenza targets. Other CMs are involved in more specific processes during the influenza infection process. Figure S3 shows the detailed information about the 100 top-ranked CMs through a circular heatmap representation of the enrichment for the DEG signatures, GO/MSigDB functions as well as influenza, inflammation, and innate immunity gene sets (Table 3).

**Table 3:** The top ranked consensus modules based on the enrichment of various differentially expressed gene sets

Module	Consensus	size	GO.BP.term	Function
Turquoise	7	689	viral reproduction	Influenza life cycle
Plum	1	668	mitochondrial RNA metabolic process	A6b1 and A6b4 integrin pathway
Gray16	1	800	organelle organization	AURORA A pathway
Coral	1	1297	organelle organization	Telomerase pathway
Green2	1	644	single-organism cellular process	MAPK targets nuclear events mediated by MAP kinases
Darkgoldenrod3	1	340	negative regulation of intrinsic apoptotic signaling pathway	p38 gamma delta pathway
Darkorchid3	1	297	lipid metabolic process	TAP63 pathway
Gray14	1	330	NK T cell proliferation	Golgi associated vesicle biogenesis
Cornsilk4	1	209	transcription, DNA-dependent	Generic transcription pathway
Cyan	1	666	regulation of timing of cell differentiation	Developmental biology
Firebrick	1	265	ventricular zone neuroblast division	RNA degradation
Darkseagreen1	1	320	hydrogen peroxide catabolic process	Activation of the mRNA upon binding of the cap binding complex and eIFs and subsequent binding to 43S
Black	1	654	response to external stimulus	Acyl chain remodelling of pc

Meta-analysis of connectivity in the co-expression networks of the 12 data sets, by summing *log*_*10*_ *(network connectivity)* obtained from the weighted co-expression networks, identified *MDM2* (MDM2 proto-oncogene) and *DOCK5* (dedicator of cytokinesis 5) as the top two most connected genes among the SRGs (see Table S3). Both *DOCK5* and *MDM2* are differentially expressed in 9 of the 12 data sets.

**Fig. 3:**
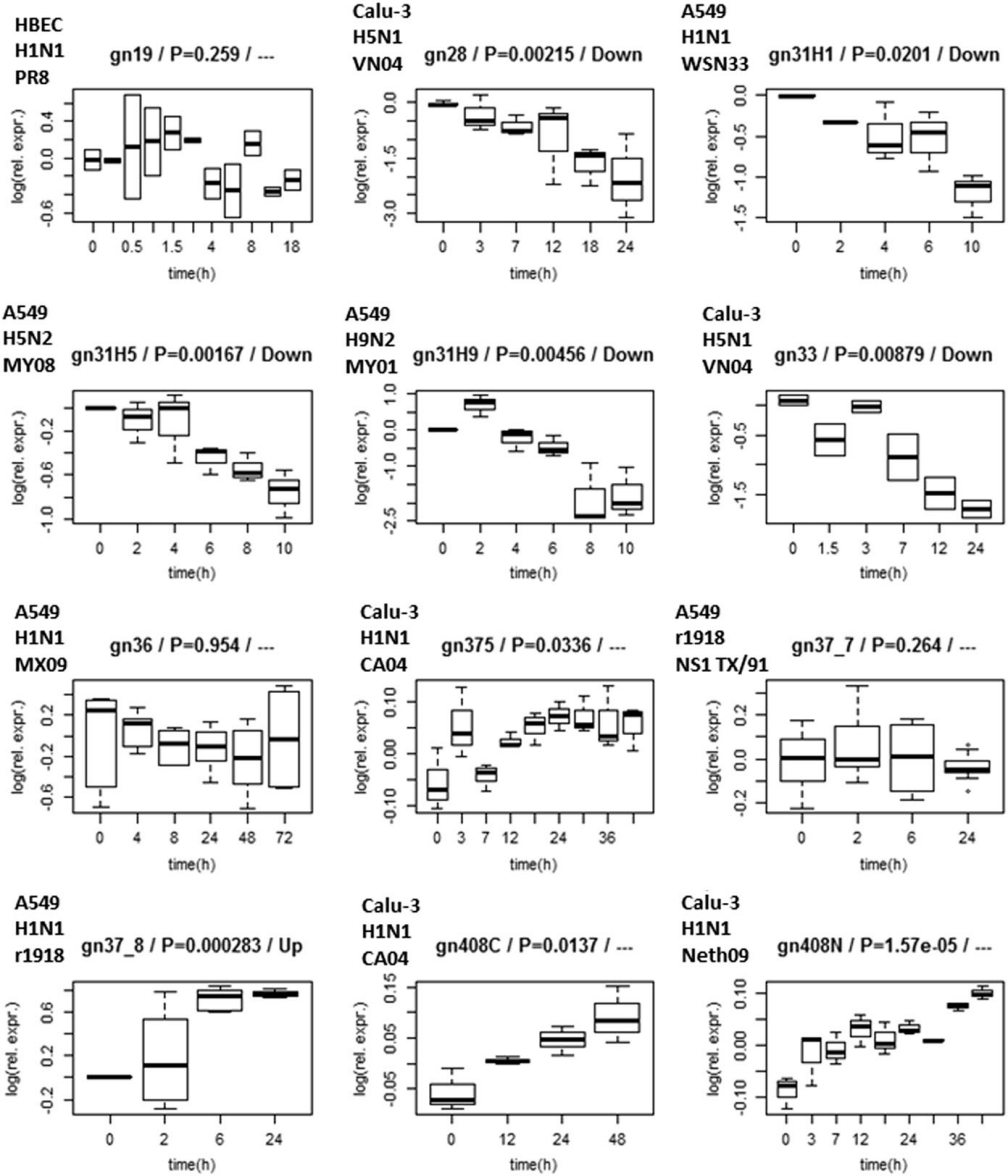
Expression profiles of *DOCK5* in 12 data sets. The time-series responses of *DOCK5* in the 12 data sets are shown together with the adjusted *p*-value after ANOVA time series analysis and significant trend after Jonckheere trend analysis. *DOCK5* can be observed to be significantly up-regulated in 1 and down-regulated in 5 data sets - “Significant” is defined to be statistically significant after Jonckheere test and requiring an overall fold change of 1.2 or greater between measurements at time *t = *0 day and the last time-point in the time-series

While the functions of *MDM2* have been well studied (it was mentioned by 7312 papers in PubMed), very little is known about *DOCK5’s* function in general (only 35 papers about *DOCK5* in PubMed). P53 modulation by *MDM2* during viral infection has been reported in the literature. *MDM2* and p53 polymorphisms were show to be associated with the development of hepatocellular carcinoma in patients with chronic hepatitis B virus infection.^36^ Furthermore, the *MDM2*-dependent inhibition of p53 is required for Epstein-Barr virus B-cell growth transformation and infected cell survival.^37^ With respect to IAV infection, the accumulation of p53 in IAV infected cells is due to the stabilization of p53 associated with compromised *MDM2*-mediated ubiquitination of p53.^38^ Jonckheere trend analysis reveals that *MDM2* is up-regulated in 5 data sets and down-regulated in 4, whereas *DOCK5* is up-regulated in 1 data set and down-regulated in 5 data sets (Fig. 3). In comparison, typical host defense genes, such as chemokine *CCL5*, interferon α inducible protein 27 (*IFI27*), *OAS2*, and *IRF7* are up-regulated in the majority of the data sets. In contrast, known host factors, such as *NXF1* (up-regulated and down-regulated in 3 data sets and 1 data set, respectively), *COPA* (up-regualted and down-regulated in 2 and 5 data sets, respectively), or *SF3B1* (up-regulated and down-regulated in 2 data sets and 1 data set, respectively) show similar diverse expression across the data sets (Table S3). Thus, a simple expression analysis is insufficient to decipher the functional role of host factors during the influenza life cycle.

### A *DOCK5-*centered network captures key biological processes in influenza infection

As WGCNA does not generate actual networks, we explicitly constructed *DOCK5*-centered unweighted co-expression networks for comparison with *DOCK5 knockout* (*DOCK5-*ko) signatures. To do this, we first identified the genes significantly (FDR < 0.05) correlated with *DOCK5* in each of our 12 assembled data sets, and then defined the consensus correlations conserved across at least 7 of the data sets (Table S4). The genes significantly correlated with *DOCK5* in at least *n* data sets are termed *DOCK5-*correlated consensus gene set, *DOCK5-*CCGS(*n*), where *n* = 2, 3, …, 12. Table S4 shows the sizes of these *DOCK5-*CCGS with corresponding genes listed in Table S5. Eight genes are correlated with *DOCK5* in 11 data sets, including abhydrolase domain containing 2 (*ABHD2*), acetyl-CoA acyltransferase 2 (*ACAA2*), *CD47* molecule, DEAD-box helicase 17 (*DDX17*), karyopherin subunit alpha 4 (*KPNA4*), tumor suppressor protein neurofibromin 2 (*NF2*), *RuvB* like AAA ATPase 1 (*RUVBL1*), and the trans-golgi network protein 2 (*TGOLN2*). *ABHD2* was shown to be important in Hepatitis B virus propagation.^39^ *DDX17* was shown to promote production of HIV-1 particles,^40^ facilitate viral RNA synthesis in H5N1 infection of human cells,^41^ and regulate alternative splicing.^42^ *KPNA4* is an importin and docks proteins with NLS signals to the nuclear pore complex. *NF2* coordinates collective migration of epithelial cells.^43^ *RUVBL1* regulates the Fanconi anaemia core complex.^44^

To understand the functions of *DOCK5* in influenza infection and defense, we performed a comprehensive functional analysis of the *DOCK5-*centered network conserved in a majority of the data sets, i.e., *DOCK5-*CCGS(7), through enrichment tests of known pathways and relevant gene signatures, as well as the previously identified differentially expressed gene sets including SRGs(7) and JTGs(7). We distinguished JTGs(7) with up-trends, i.e., JTGs^up^(7), from those with down-trends (JTGs^down^). We considered the following gene signatures in addition to functional GO and MSigDB genes sets: (i) two sets of influenza host factors derived from siRNA data^5,21^ (sets *i*_a_ and *i*_b_, respectively), (ii) a set of “inflammasome” genes,^22^ (iii) a set of host defense genes relevant for innate immunity,^24^ and (iv) a set of interferon-stimulated genes.^23^ *DOCK5-*CCGS(7) is enriched for a number of pathways including the ER-nucleus signaling, response to ER stress, RNA localization, Golgi vesicle transport, viral process, modulation by virus of host morphology, RNA splicing, and cellular protein metabolic process (Table S6). The 985 genes shared by *DOCK5-*CCGS(7) and SRGs(7) (FET *p* < 1e−320, 4.3-fold) are involved in processes required for both the viral life cycle and antiviral host-defense. *DOCK5-*CCGS(7) and JTGs^up^(7) share 226 genes (FET *p* = 6.2e−76, 3.5-fold) that are associated with interferon signaling and host response to virus, while the 762 genes shared by *DOCK5-*CCGS(7) and JTG^down^(7) (FET *p* = 1.1e−239, 3.2-fold) are involved in lipid and fatty acid metabolism (Tables S7 and S8).

Known influenza host factors are enriched in *DOCK5-*CCGS(7) (FET *p* = 0.035, 1.6-fold) (Table S9). The inflammasome signature is significantly enriched in *DOCK5-*CCGS(7) (FET *p* = 3.6e-5, 1.2-fold), SRGs(7) (FET *p* = 1.2e−8, 1.4-fold) and JTGs^down^(7) (FET *p* = 5.4e−7, 1.4-fold) (Table S10). The innate immune system-related genes curated by ImmuneDB are significantly enriched in *DOCK5-*CCGS(7) (FET *p* = 9.3e−10, 1.4-fold), SRGs (FET *p* = 5.6e−12, 1.7-fold), JTGs^up^(7) (FET *p* = 2.7e−15, 2.6-fold), but not in JTG^down^(7), indicating the typical activation of innate immune response. All non-empty intersections between *DOCK5-*CCGS(7), SRGs(7), and JTGs^up^(7) are also enriched for the ImmuneDB genes (Table S11). However, the down-regulated innate immune system genes from the intersection of *DOCK5-*CCGS(7) and JTGs^down^(7) (FET *p* = 0.05, 1.4-fold change) indicate potential modulation of the immune system by *DOCK5* (see next section). *DOCK5-*CCGS(7) and SRGs(7) are significantly enriched for interferon stimulated genes (ISGs) with FET *p* = 2.3e−20 (3.0-fold) and 5.3e−20 (2.2-fold), respectively (Table S12), suggesting interferon stimulation in the *DOCK5-*centered network and the overall response. The analysis above demonstrates that the *DOCK5-*CCGS(7) network captures many aspects of the immune response.

### *DOCK5* is a potential host factor for influenza infection

**Fig. 4:**
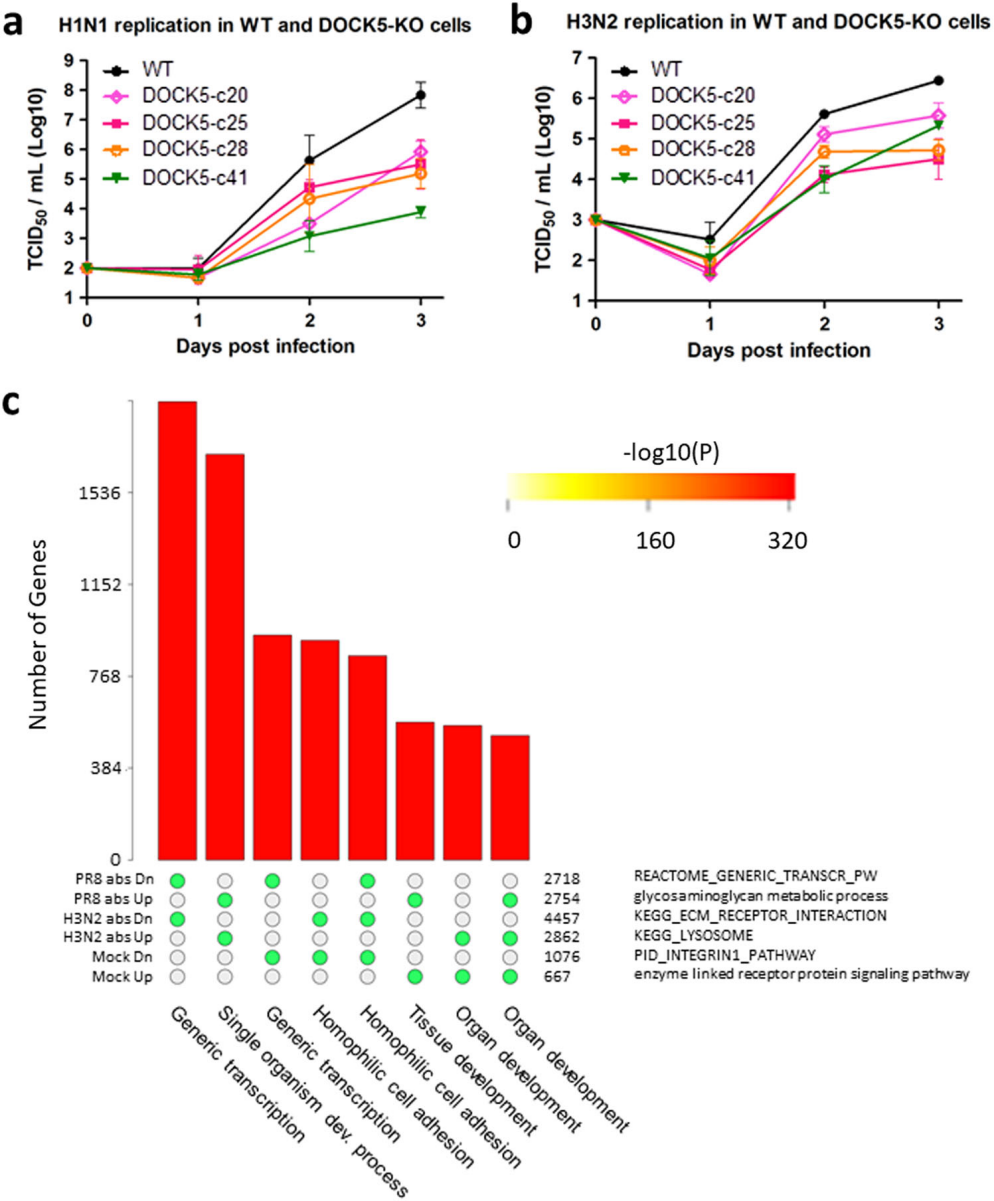
Effect of *DOCK5* knockout on the replication of influenza virus in A549 cells. H1N1 virus (**a**) and H3N2 (**b**) virus were inoculated at MOI of 0.0005 TCID_50_/cell and 0.005 TCID_50_/cell, respectively, at day 0. The replication of the virus in the wild type and *DOCK5* knockout A549 cells was determined by titration of the culture supernatants over 3 days using a TCID50 assay. Error bars represent standard deviation (SD). **c** Intersections among six selected *DOCK5-*ko DEG signatures are shown. The matrix of solid and empty circles at the bottom illustrates the “presence” (solid green) or “absence” (empty) of the gene sets in each intersection. The numbers to the right of the matrix are set sizes. The colored bars on the top of the matrix represent the intersection sizes with the color intensity showing the *p*-value significance. The intersections are ordered by *p*-values of the super exact test. The corresponding function for each gene set was also shown at the bottom and on the right hand-side of the matrix

**Fig. 5:**
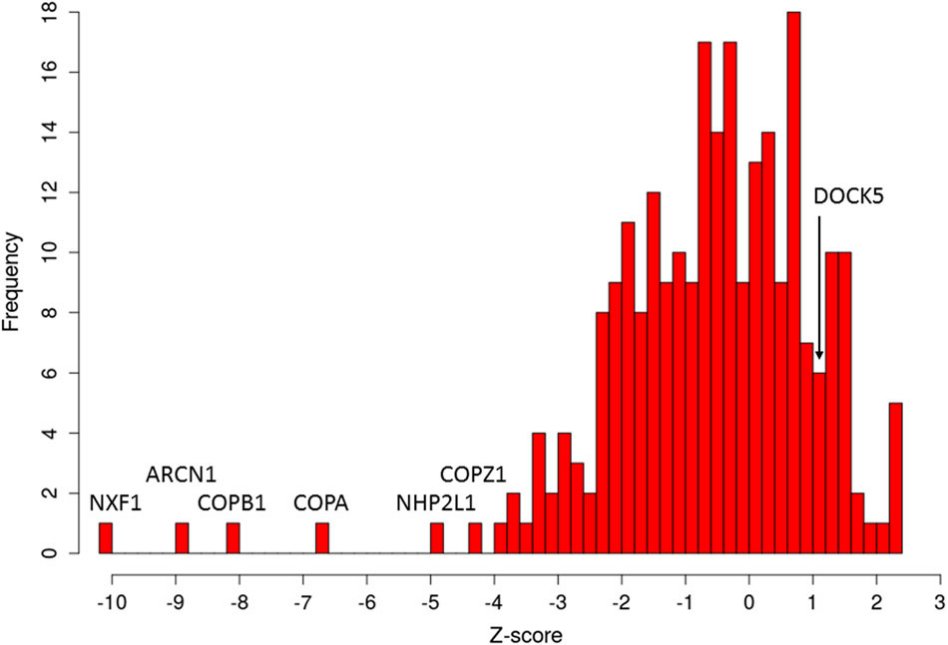
Distribution of influenza host factors after siRNA screens. A set of 254 siRNA-based host-factors^5,21^ have been used to assess their effect on the influenza virus life cycle. Corresponding *Z*-scores have been scaled and combined. Host-factors with the lowest *Z*-scores, e.g., *NXF1*, induce most severe effect on viral replication after siRNA knock-down

To validate the functional role of *DOCK5* during influenza infection, we knocked out *DOCK5* in human lung epithelial A549 cell lines using the CRISPR/Cas9 genome editing system.^45^ Virus replication in these knockout cell lines was compared to that in the wild type parental A549 cell lines. Four *DOCK5-*ko A549 clones (*DOCK5-*c20, c25, c28, and c41) were selected and compared to the wild type A549 (A549-wt) for their capacity to support the replication of influenza virus. The cells were infected with an H1N1 virus (A/Puerto Rico/8/1934) or an H3N2 virus (A/New York/238/2005) and the viral titers at days 1, 2, and 3 post infection were determined using a TCID_50_ assay. The influenza viruses replicated to significantly lower titers in *DOCK5-*ko cells than in the parental A549 cell line (Fig. 4a, b). At day 1, IAV replication in the *DOCK5-*ko cells was statistically indistinguishable from replication in *DOCK5-*wt. At day 2, the absence of functional DOCK5 protein resulted in a 20-fold decrease (or 1.30 log_10_ reduction) of the H1N1 viral titer (8.12–363.08 fold decrease corresponding to 0.91–2.56 log_10_ reduction in the 4 *DOCK5-*ko cell lines compared to A549-wt) and an 8.51 fold decrease (or 0.93 log_10_ reduction) of the H3N2 viral titer (3.16–40.73-fold decrease corresponding to 0.50–1.61 log_10_ reduction in the 4 *DOCK5-*ko cell lines compared to A549-wt). At day 3, the effect of *DOCK5* knockout was even more pronounced: the H1N1 viral titer was reduced by 436.51-fold or 2.64 log10 reduction (81.28–8709.64-fold decrease corresponding to 1.91–3.94 log_10_ reduction in the 4 *DOCK5-*ko cell lines) and the H3N2 viral titer decreased by 52.48-fold or 1.72 log10 (7.41–87.10-fold decrease corresponding to 0.87–1.94 log_10_ reduction in the 4 *DOCK5-*ko cell lines compared to *DOCK5-*wt). A hierarchical linear model (hLM) was employed using both the time past infection and wt/clone information as parameters. All 4 *DOCK5-*ko clones showed significantly different time series responses compared to the wild type cells (hLM *p*-values of range between 5.3e−7 and 5.7e−3; Table S13). Similar to some known host factors, *DOCK5* moderately compromises influenza replication when using siRNA depletion (Fig. 5). The compromised replication of both the H1N1 virus (Fig. 4a) and the H3N2 virus (Fig. 4b) in the absence of functional DOCK5 protein indicates that *DOCK5* is a potential host factor involved in the life cycle of influenza virus.

Since the parental A549 cell line used for CRISPR/Cas9 knockout is composed of a heterogeneous population of cells, the clonal variability of the selected *DOCK5-*ko clones (*DOCK5-*c20, -c25, -c28, and -c41) may have resulted in the variable, though significantly reduced capacity of these cells to support the replication of influenza viruses (Fig. 4a, b). *DOCK5-*c28, whose cellular morphology and proliferation rate are closest to the A549-wt cells, was chosen as a representative clone to determine the effects of knockout perturbation on host RNA responses (Fig. 4c). Real-time qPCR was used to further quantify the expression level of some important genes in the wild type and *DOCK5-*c28 A549 cells, under infection conditions of Mock, H1N1, and H3N2.

### The *DOCK5-*regulated transcriptome involves cytokinesis, vesicle trafficking, and splicing

The transcriptional program regulated by *DOCK5* was characterized by sequencing mRNA from the cells of the representative clone *DOCK5-*c28 under different conditions as combinations of infection status (H1N1, H3N2, or Mock infection) and *DOCK5* perturbation status (with or without *DOCK5-*ko). These data sets are referred to as the “validation data set”. Differential expression analysis was performed on the following “contrasts” using Bioconductor’s limma package: relative *DOCK5-*ko vs. wild type (the relative expression of a gene was the ratio of the gene’s expression in H1N1/H3N2 infection to that in the corresponding MOCK), and absolute *DOCK5-*ko vs. wild type (the absolute expression values in H1N1, H3N2, and MOCK infections were used). A fold change cutoff of 1.2 and an FDR threshold of 5% were employed to identify the corresponding DEG signatures.

Without influenza infection (i.e., MOCK infection), *DOCK5* predominantly modulates cell migration, as indicated by the significant enrichment of cell receptor signaling (FET *p* = 1.3e−8, 2.4-fold), cell communication (4.9e−4, 1.3-fold), cell migration (FET *p* = 6.1e−3, 1.9-fold), extracellular matrix receptor interaction (FET *p* = 8.6e−3, 2.8-fold), and integrin pathways (FET *p* = 2.5e−2, 3.8-fold) in the DEG signature in the MOCK *DOCK5-*ko cells in comparison with the MOCK wildtype cells (Tables S14 and S15). Similarly, enriched pathways can be observed in the case of infection (Tables S16–S18). During infection, pathways that are up-regulated by the knockout of *DOCK5* involve carbohydrate metabolism (FET *p* = 3.3e−4, 1.5-fold), in particular aminoglycan processes (FET *p* = 2.4e−3, 2.2-fold), lysosome functions (FET *p* = 5.8e−3, 2.1-fold), and phospholipid metabolism (FET *p* = 2.0e−2, 1.9-fold). Whereas down-regulated pathways in *DOCK5-*ko conditions compared to *DOCK5-*wt include cell adhesion (FET *p* = 1.0e−6, 1.6-fold), extracellular matrix receptor interaction (FET *p* = 9.4e−6, 2.8-fold), and integrin pathways (FET *p* = 3.0e−3, 2.5-fold). After removing genes responding to MOCK infection from the *DOCK5-*ko DEG signatures in the case of infection, we performed enrichment tests of functional pathways. *DOCK5* knockout induces downregulation of cell adhesion (FET *p* = 2.7e−13, 1.7-fold), GPCR ligand binding (FET *p* = 6.1e−9, 2.2-fold), GPCR signaling (FET *p* = 5.2e−7, 1.9-fold), and extracellular matrix receptor interaction (FET *p* = 2.8e−6, 2.7-fold). However, no pathways were enriched in the case of upregulated genes by *DOCK5-*ko (Tables S19 and S20).

Figure 4c shows the highly significant overlap among these DEG signatures of *DOCK5-*ko (Tables S21 and S22 for details). The genes up-regulated by *DOCK5-*ko in both H1N1 and H3N2 infections are enriched for enzyme linked receptor protein signaling, cell communication, cell adhesion, and cell migration pathways; whereas the down-regulated genes are involved in single-multicellular organism process, generic transcription, and integrin pathways. Given the highly significant overlap between these DEG signatures under the H1N1 and H3N2 infections, we focus on the signature observed under H3N2 infection although results for all DEG signatures are presented in the supplementary documents. The 2863 up-regulated genes induced by *DOCK5-*ko under the H3N2 infection are termed sg*DOCK5-*DEG+, while the 4512 down-regulated genes are called sg*DOCK5-*DEG.

The striking conservation of the expression of hnRNPs and *SYNCRIP* across 11 studies suggests that splicing is a significant process during influenza infection. To further investigate this process, we evaluated differential exon splicing and exon usage based on the RNAseq data from the samples in the *DOCK5-*ko experiments. The number of genes that displayed differential exon usage ranges between 13 and 2851, depending on the specific scenario (Table S23). Among all the different scenarios, the cysteinyl-tRNA synthetase 2 (*CARS*), glucosyltransferase ALG5, and non-imprinted in Prader-Willi/Angelman Syndrome 1 (*NIPA1*) showed the most significant different exon usage. There are 137 exon variants in a collagen gene *COL7A1*, 118 exon variants in high density lipoprotein binding protein (*HDLBP*), and 112 exon variants in microtubule-actin crosslinking factor 1 (*MACF1*). *COL7A1* is responsible for anchoring epithelial cells to the stroma, *HDLBP* is known to support cell proliferation, and *MACF1* couples the microtubule network to cellular functions. Despite a small snapshot, these three top-ranked genes do suggest a role for *DOCK5* in cytokinesis. In the MOCK infection, genes and corresponding pathways relevant for splicing are subject to differential splicing/exon usage (Table S24). H3N2 infection induces differential exon usage in pathways responsible for cellular macromolecular metabolic processes, i.e., protein modification/protein phosphorylation/signaling as well as regulation of apoptosis (Tables S25 and S26). A weaker, but nevertheless significant, differential exon usage can be observed during H1N1 infection with respect to viral process pathways and pathways responsible for virus-induced modulation of host morphology and physiology (not shown).

### Validation of the *DOCK5-*centered network

**Fig. 6:**
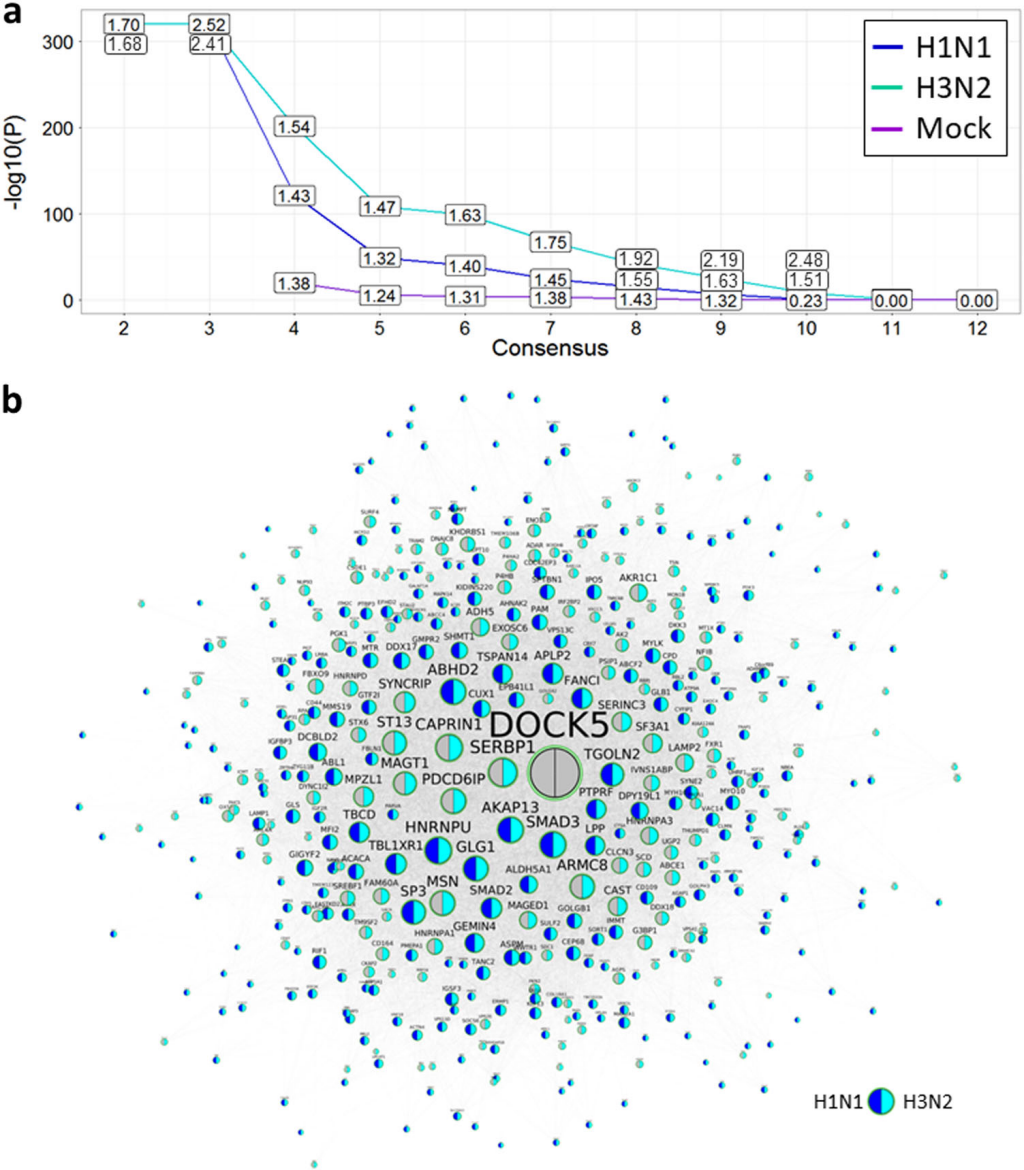
Validation of *DOCK5* centered gene co-expression networks (*DOCK5-*CCGS) by the gene signatures (sg*DOCK5-*DEGS) identified from the *DOCK5* knockout experiments. **a** The enrichment of the *DOCK5* centered network for the differentially expressed gene sets induced by the knockout of *DOCK5* under H1N1, H3N2, and MOCK infections. The x-axis shows the *DOCK5-*centered co-expression network *DOCK5-*CCGS(n) conserved in at least n data sets, *n* = 2,3,…,12. The y-axis indicates the—log_10_ of corrected FET *p*-values. Boxed labels show corresponding fold-changes. **b** A subnetwork of *DOCK5-*CCGS(7), i.e., the genes correlated with *DOCK5* in at least 7 data sets shows the genes shared by *DOCK5-*CCGS(7), *DOCK5’s* knockout signature (sg*DOCK5-*DEG^+^) and the union of the consensus differential expression gene signature SRGs(7) and the consensus down-regulated gene signature JTGs^down^(7). The selected nodes are up-regulated by *DOCK5* in either the H1N1 or H3N2 infection. The left and right color sectors in each node indicate whether it was differentially expressed in H1N1 infection (*blue* for significant differential expression and grey for non-significant one) and H3N2 infection (*cyan* for significant differential expression and grey for non-significant one), respectively

We validated the previously identified consensus modules and *DOCK5-*centered networks (*DOCK5-*CCGS(n)) by the DEG signatures identified from the *DOCK5* knockout experiments (sg*DOCK5-*DEGS) to achieve an in-depth understanding of the biological functions of *DOCK5* (Fig. 6). This analysis was done for the DEG signatures from 15 configurations (Fig. S5 and S6; Table S27) a detailed discussion was included in the Supplemental Information. Among 282 consensus modules, 5 are significantly enriched for the genes up-regulated or down-regulated by *DOCK5* during MOCK infection, 27 for the genes upregulated by *DOCK5* knockout during H1N1 infection, 31 for the genes upregulated by *DOCK5* knockout during H3N2 infection, 7 for the genes down-regulated by *DOCK5* knockout during H1N1 infection, and 16 for the genes down-regulated by *DOCK5* knockout during H3N2 infection. Eleven of the top 13 modules in Table 3 are significantly enriched for sg*DOCK5-*DEGS. These modules have different functions, such as viral reproduction, single organism cellular process, and organelle organization, indicating that *DOCK5* regulates a diversity of biological processes during IAV infection.

**Fig. 7:**
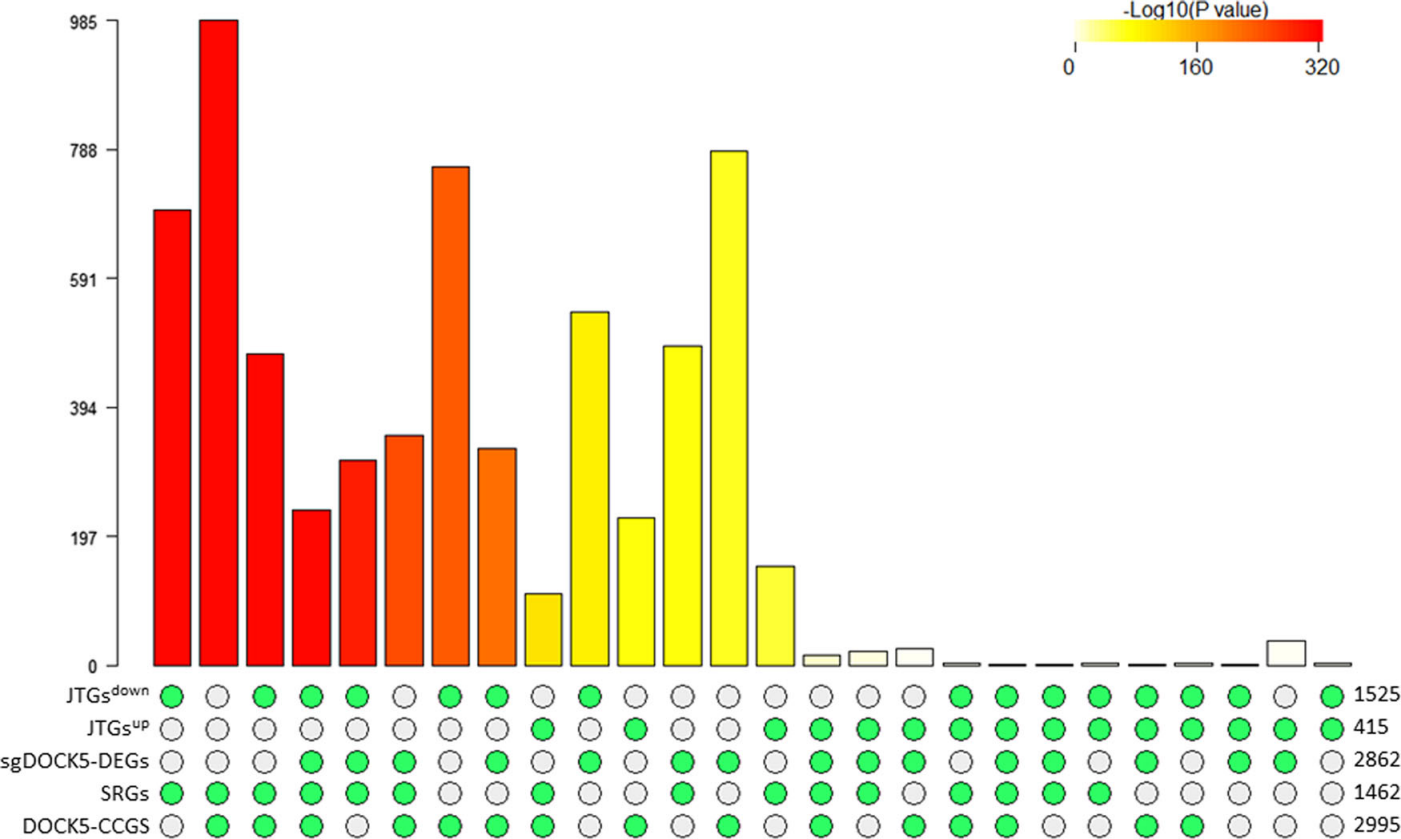
Intersections amongst *DOCK5-*CCGS(7), SRGs(7), JTGs^up^(7), JTGs^down^(7) and sg*DOCK5-*DEG^+^ by Super Exact Test. The bar chart plot shows the combinations of the five gene sets with non-empty intersections. The matrix of solid and empty circles at the bottom illustrates the “presence” (solid green) or “absence” (empty) of the gene sets in each intersection. The numbers to the right of the matrix are set sizes. The colored bars on the top of the matrix represent the intersection sizes with the color intensity showing the *p-*value significance. The intersections are ordered by *p*-values of the super exact test

Highly significant overlap among *DOCK5-*CCGS(7), SRGs(7), JTGs(7), and sg*DOCK5-*DEG^+^ by the Super Exact Test (SET)^46^ strongly validated our predicted centered network, as shown in Fig. 7 (see also Fig. S4 and S7). The intersection of *DOCK5-*CCGS(7), SRGs(7), and sg*DOCK5-*DEG^+^ includes 24 interferon-stimulated genes, i.e. ISGs (Table S28). Among the 24 genes, only *ISG20*, *IL6*, and *STAT1* are predominantly up-regulated across the data sets while all other genes are either up-regulated or down-regulated in comparable numbers of data sets. *ISG20*, an antiviral ribonuclease required for viral replication,^47^ shows a large difference in expression between the *DOCK5-*wt cells and *DOCK5-*ko cells. *ISG20* was up-regulated by over 8-fold 2 days post-infection. In the *DOCK5-*ko cells, the expression of *ISG20* increased to over 20-fold 2 days after post-infection.

Although *DOCK5-*CCGS(7) is not significantly enriched for sg*DOCK5-*DEG^−^ (i.e., genes that are downregulated in *DOCK5-*ko compared to *DOCK5-*wt under the H3N2 virus infection), the genes in their intersection are involved in regulation of transcription and phosphate metabolic processes. Some of the most conserved up-regulated genes are in the overlap between *DOCK5-*CCGS(7) and sg*DOCK5-*DEG^-^ and they include the vesicle associated membrane protein 5 (*VAMP5*), as well as cytokines. *VAMP5* is a member of the VAMP/synaptobrevin family and the SNARE superfamily, and may participate in vesicle trafficking events potentially (indirectly) controlled by *DOCK5. VAMP5* is significantly upregulated in *DOCK5-*wt cells. Under *DOCK5-*ko conditions, upregulation of *VAMP5* is significantly reduced. Furthermore, *VAMP5* is a curated target of the *JUN* transcription factor. Thus, *VAMP5* expression could be potentially modulated by *JUN*, alternatively under direct control of *DOCK5*. The observed restriction of up-regulation in the *DOCK5-*ko cells compared to the wild type may indicate a decline in host defense response. As a functional DOCK5 is absent in *DOCK5-*ko, so are the *DOCK5-*dependent processes during influenza infection. Therefore expression of the predominately host defense genes (such as *CXCL11*, *IRF7*, or *JUN*) may no longer be required at a high transcriptional level. According to the ENCODE data, *JUN*, as a transcription factor, could potentially regulate *DOCK5*.^48^ In the RNAseq experiments, *JUN* itself is up-regulated in *DOCK5-*wt and not in the *DOCK5-*ko cells. Data from the library of integrated network-based cellular signatures (LINCS, see Supplemental Information for details) indicates a feedback between *JUN* and *DOCK5*, with *JUN* down-regulating *DOCK5* as transcription factor and *DOCK5* (indirectly) activating *JUN*.

The overlap between *DOCK5-*CCGS(7) and the sg*DOCK5-*DEGs consists of genes such as *TGOLN2*. As discussed above, *TGOLN2* is significantly correlated with *DOCK5* in 11 out of 12 data sets. The genes correlated with *TGOLN2* within the *DOCK5* neighborhood include golgins such as *GOLGA2*, *GOLGA4*, *GOLGB1*, *GLG1*, and *GOLPH3*. Proteomic analysis shows that *GLG1* binds to the influenza virus *PB1-F2* protein^49^ though the consensus siRNA screening result indicates it does not function as a host factor during the influenza virus life cycle.^50^ The intersection of *DOCK5-*CCGS(7) and the sg*DOCK5-*DEG^+^ also includes *AP1S1*, which is part of the clathrin coat assembly complex linking clathrin to receptors in coated vesicles. These vesicles are involved in endocytosis and Golgi processing. The *DOCK5* network neighbors also include a member of the lysosomal ATPases (*ATP6V1A*) and *COPG2*, one of the 7 subunits of the coatomer 1 vesicular transport complex (COPI). The lysosomal (or vacuolar) ATPase complex was suspected to be required for influenza vesicle entry.^14^ A similar assessment has been made for COPI. Presumably, *DOCK5* utilizes *COPG2* as an entry point for COPI modulation.

Another process potentially modulated by *DOCK5*, is mRNA processing, in particular splicing. Prominent members of the spliceosome include *hnRNPA1*, *hnRNPD*, and *SYNCRIP* that are all commonly expressed as SRGs in almost all data sets, as discussed above. These 3 genes are correlated with *DOCK5* in at least 8 data sets. *SYNCRIP* is a host factor that was shown to be involved in hepatitis C virus RNA replication,^31^ and required by the HCV IRES for translation-competent 48S complex formation.^32^ It was also previously found to be associated with immune functions.^33^ Other genes in the intersection between *DOCK5-*CCGS(7) and sg*DOCK5-*DEG^+^ are the Influenza virus NS1 binding protein (*NS1BP, IVNS1ABP*, or *hnRNP-I*) and splicing factors 3 (*SF3A1*, *SF3A3*, and *SF3B1*). *NS1BP* is a required viral host factor relevant for pre-mRNA processing, mRNA metabolism, and transport.^5^ It is also a key mediator of IAV gene expression, in particular viral RNA splicing.^51^ Furthermore, *NS1BP* modulates tumor suppressor, and potential host defense gene sirtuin 3 (*SIRT3*), which is down-regulated during infection in 9/12 data sets (with no significant directional change in expression in the remaining 3 data sets). The exosome component 6 (*EXOSC6*) is another member in the overlap that is differentially expressed in 9/12 data sets and significantly correlated with *DOCK5* in 10/12 data sets. The exosome controls alternative splicing by mediating the gene expression and assembly of the spliceosome complex.^52^ *EXOSC6*, *hnRNPs*, *NS1BP*, and the splicing factors form a tight sub-network within *DOCK5-*CCGS(7). Nuclear pore proteins assemble an additional class of genes that are required for splicing/mRNA processing, as they are responsible for the transport of vRNPs into the nucleus. Our data show that *NUP35*, *NUP50*, *NUP93*, *NUP133*, and *NUP210* are correlated with *DOCK5*.

The potential overall effect of *DOCK5-*ko on general cellular functions such as splicing has been evaluated by analyzing functional processes of the corresponding DEGs between the relative expression in *DOCK5-*wt vs. *DOCK5-*ko during H1N1/H3N2 infections, and the relative expression in *DOCK5-*wt vs. *DOCK5-*ko during MOCK infections (Tables S29 and S30). Disregarding directional DEG responses, functional processes involve multicellular organismal processes, extracellular matrix and structure organization, cell adhesion, and cell–cell signaling.

Pathways that are up-regulated in the *DOCK5-*wt scenario, i.e., pathways that we have identified to require *DOCK5* functionality, involve extracellular matrix receptor interaction, GPCR ligand binding, and unfolded protein response. Whereas, pathways that are down-regulated, i.e., activated pathways after *DOCK5-*ko, are cellular glucuronidation, fibroblast apoptotic process, and cellular response to xenobiotic stimulus. These enriched functions may indicate a potential breakdown of cellular integrity due to lack of *DOCK5* functionality. Although such a breakdown of cellular integrity can also be caused by viral infection and induced cell death, we are confident that such a scenario and the potential influence on the identified role of *DOCK5* can be excluded. First, validation experiments and data sets with intermediate to high multiplicity of infections (MOIs) were used, the latter well controlled due to the consensus approach. Second, the identified effects between *DOCK5* and target genes are correlation-based and the causal role of *DOCK5* in this process has been identified in combination with the *DOCK5-*ko experiments, as described. A simple difference in proliferation/cell death would not display such a causal pattern with *DOCK5* as center. Furthermore, pathways relevant for apoptotic processes have been observed in *DOCK5-*CCGS(7) as discussed above, but not in overall significantly expressed genes, such as SRGs(7) or JTGs(7) (see Table S7). Also, apoptotic pathways were only identified as being significantly expressed between *DOCK5-*wt and *DOCK5-*ko scenarios (Tables S29 and S30). Thus, the causal role of *DOCK5* in these processes can be established and potential effects caused by virus-induced cell death ruled out.

Given the information on differential exon splicing/usage from the *DOCK5-*ko RNAseq data, genes and processes that show differential splicing effects induced by *DOCK5* were further investigated. We specifically evaluated the overlap between *DOCK5-*induced differentially spliced genes and the *DOCK5* network neighborhood (Table S31). *DOCK5-*CCGS(7) is most significantly enriched for the gene set of differential exon usage after H3N2 infection (FET *p* = 1.43e−119, 2.0-fold) and their overlap is associated with protein modification, signal transduction, regulation of localization, adherence junction, and regulation of the SMAD2/3/4 transcriptional activity (Tables S32 and S33). In particular, the overlap is enriched for influenza virus host factors,^5^ indicating that *DOCK5* may be required for the regulation of mRNA processing and splicing of genes relevant for the influenza life cycle. We further explored if and how gene splicing affected by influenza infection differs from gene splicing potentially induced by *DOCK5-*modulated splicing processes. Differential splicing between influenza-infected and mock-infected cells were compared with or without the perturbation of DOCK5. *DOCK5-*CCGS(7) is enriched for the differentially spliced gene signatures (WT: H3N2 vs Mock, FET *p* = 1.1e−24, 2.2-fold change; *DOCK5-*ko: H3N2 vs. Mock, FET *p* = 5.8e−9, 2.0-fold change), the intersections are not enriched for influenza host factor signatures or the inflammasome. Furthermore, none of the original differentially spliced gene sets show any significant overlap with influenza host factor genes or inflammasome genes. Therefore, *DOCK5* is likely to be required for proper splicing, in particular of host genes relevant for the influenza life cycle.

**Fig. 8:**
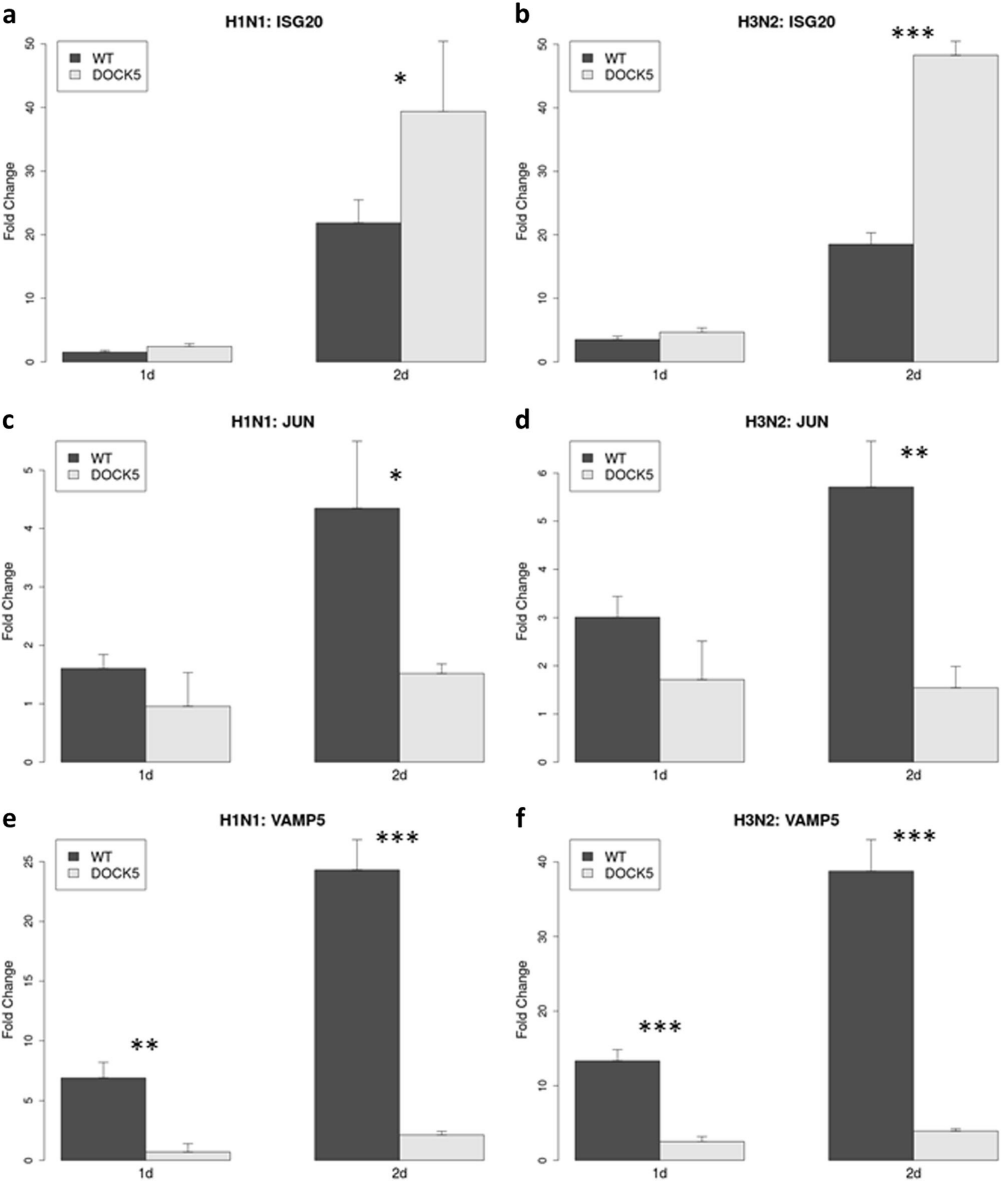
Validation of the changes of expression of *DOCK5* key targets under different scenarios as combinations of the *DOCK5* perturbations (*DOCK5-*wt and *DOCK5-*ko) and virus infections (H1N1 and H3N2 infections) by RT-qPCR at day 1 and day 2 post infection. The expression of *ISG20* (**a**, **b**), *JUN* (**c**, **d**) and *VAMP5* (**e**, **f**) was measured by RT-qPCR during H1N1 (**a**, **c**, **e**) and H3N2 (**b**, **d**, **f**) infections. Difference in expression of each gene between *DOCK5-*wildtype (WT) and *DOCK5-*ko (*DOCK5*) during either H1N1 or H3N2 infection was assessed by *t*-test. Significant differences were indicated by *(*p* < 0.05), **(*p* < 0.01) and ***(*p* < 0.005)

In order to verify the role of *DOCK5* in modulating key cellular processes predicted by the network analysis and confirmed by the RNAseq data, we further measured mRNA levels of highly responding genes in *DOCK5-*CCGS(7) using real-time quantitative PCR (RT-qPCR). The genes were selected based on the transcriptome sequencing data (requiring strong transcriptional response, i.e., significant difference between *DOCK5-*wt and *DOCK5-*ko cells) and network analyses. These genes include upstream transcription factor *JUN* that may influence *DOCK5* expression and two potential downstream targets of *DOCK5* (*ISG20* and *VAMP5*) representing examples of *DOCK5-*mediated processes such as immune system quenching (*ISG20/JUN*) and vesicle transport (*VAMP5*). Their expression profiles are similar to the *DOCK5-*c28 transcriptome data, but significantly different from that in the wild type A549 cells (Fig. 8). For *ISG20*, its upregulated expression during both H1N1 and H3N2 influenza infection in mRNA level was significantly enhanced in *DOCK5-*ko cells compared to *DOCK5-*wt cells (Fig. 8a, b). At day 1, neither *ISG20* nor *JUN* showed significant difference between *DOCK5-*wt and *DOCK5-*ko cells during the infections but *VAMP5* was already significantly down-regulated by *DOCK5-*ko (H1N1: *p* = 0.0056, 0.1-fold change; H3N2: *p* = 0.0020, 0.19-fold change). At day 2, *ISG20* was significantly up-regulated by *DOCK5-*ko (H1N1: *p* = 0.03, 1.8-fold change; H3N2: *p* = 0.0013, 2.5-fold change; Fig. 8a, b) while *JUN* and *VAMP5* were down-regulated by *DOCK5-*ko during IAV infection (Fig. 8c–f). Specifically, at day 2, *JUN* expression was down by about 70% (H1N1: *p* = 0.045, 0.35-fold change; H3N2: *p* = 0.076, 0.27-fold change) and *VAMP5* was down by about 90% (H1N1: *p* = 0.0038, 0.087-fold change; H3N2: *p* = 0.0046, 0.10-fold change) in *DOCK5-*ko cells compared to *DOCK5-*wt cells.

We have further evaluated the potential effect of influenza proteins on *DOCK5* and identified cellular functions relevant for the influenza life cycle. For this purpose, we used experimentally obtained protein–protein interaction data of influenza protein interactions with host factors. Durmuş and Ülgen assembled pathogen–host interactions of 11 DNA virus families and 15 RNA virus families, including influenza.^53^ We extracted the influenza A specific network consisting of 11 influenza proteins and 1621 host factors. Significant overlap between human host factors interacting with 9 viral proteins and 231 targets in *DOCK5-*CCGS(7) were observed (Fig. S8 and Table S34). Among the most significantly enriched overlap, with fold enrichment of 2 or higher, are human host factors interacting with influenza proteins HA, M1, M2, NA, and NP. Although primary functions of the corresponding intersections are according to the processes modulated by the specific viral protein (e.g., the M1 protein and early phase of viral life cycle function, or ribonucleoprotein complex assembly and RNA transport with respect to NP), a majority of the functions involve mRNA processing and splicing (Tables S35 and S36). Other functions involve viral process, the proteasome, and protein localization and transport. The latter processes are indicated by host factors interacting with the viral M2 and NS1 proteins. Thus, these findings validate the significant impact of *DOCK5* on the genes discussed and corresponding pathways.

## Discussion

In this study, we systematically analyzed a large amount of gene expression data from 12 molecular studies of influenza infection covering MOIs between 0.1 and 5 (median 2.5, see Table 1). We first identified differentially expressed genes using ANOVA and then derived robust consensus DEG signatures across multiple studies. Many of those DEGs have been known to play essential roles during influenza infections. By employing non-parametric Jonckheere trend analysis we identified significant up-regulated and down-regulated genes required for viral replication or activated as a host defense. Type I interferon signaling and the up-regulation of interferon stimulated genes or ISGs, such as *MX1*, *IFITM*, *IRF7*, and *OAS*, among other ISGs, are well conserved across all 12 studies. On the other hand, down-regulated genes are associated with small molecule/lipid metabolic processes and localization. The heterogeneous nuclear ribonucleoprotein A1 (*hnRNPA1*) is the highest ranked gene significantly expressed in all 12 data sets and predominantly down-regulated. Together with splicing factor 2 (*SF2*), it regulates alternative splicing of interferon regulator factor-3 (*IRF3*).^28^ Mediated by transportin 1 (*TNPO1*),^29^ *hnRNPA1* shuttles between the nucleus and the cytosol.^30^ Other highly ranked members of the hnRNP family involve the related *hnRNPD* and the synaptotagmin binding cytoplasmic RNA interacting protein (*SYNCRIP*), both significantly expressed in 11 data sets.

To further understand the co-regulation among the genes in response to influenza infection, we performed gene co-expression analysis to identify 1191 modules from 12 studies, which were further used to derive 282 consensus co-expression modules. These consensus modules have functions from viral reproduction to *RIG-I* signaling and Golgi associated vesicle biogenesis. We formally rank-ordered the 282 consensus modules by their enrichment for the ANOA and Jonckheere Trend analysis-based DEG signatures derived from the individual studies. Two members of the top-ranked module, *MDM2* and *DOCK5*, are most connected across all 12 co-expression networks and they were predicted to be the top drivers of the gene networks and potential host factors for influenza infection. We sought to comprehensively examine the role of *DOCK5* during influenza infection. We explicitly constructed *DOCK5-*centered networks, which capture many known processes and host factors for influenza infection, including the ER-nucleus signaling, response to ER stress, RNA localization, Golgi vesicle transport, viral process, modulation by virus of host morphology and RNA splicing, as well as the cellular protein metabolic process. We validated experimentally *DOCK5* and its co-regulated networks. *DOCK5* was knocked out in human lung epithelial A549 cell lines and virus replication was compared to that in the wild type parental A549 cell lines. The influenza viruses replicated to significantly lower titers in *DOCK5-*ko cells than in the parental A549 cell line indicating impairment of viral replication without functional *DOCK5*. We also characterized the transcriptional program regulated by *DOCK5* by sequencing mRNA from infected and uninfected wild type and *DOCK5-*ko cells. Our network approach, combined with knockout data and comparative analysis between different genetic and infection scenarios, validates the causal role—be direct or indirect—of DOCK5 in these processes. Three genes, *ISG20*, *JUN*, and *VAMP5*, were selected and their expression re-validated by RT-qPCR. The co-regulatory network was validated by the DEG signatures identified from the *DOCK5* knockout experiments to achieve an in-depth understanding of the biological functions of *DOCK5*. Our results demonstrate that *DOCK5* is a host factor that is potentially required for viral replication in cell culture. We further demonstrated by our coexpression network analysis approach that *DOCK5* not only modulates processes that are important for the IAV life cycle but also potentially subverts the host defense response by directly compromising key defense genes, or by indirectly affecting cellular factors required for host defense.

**Fig. 9:**
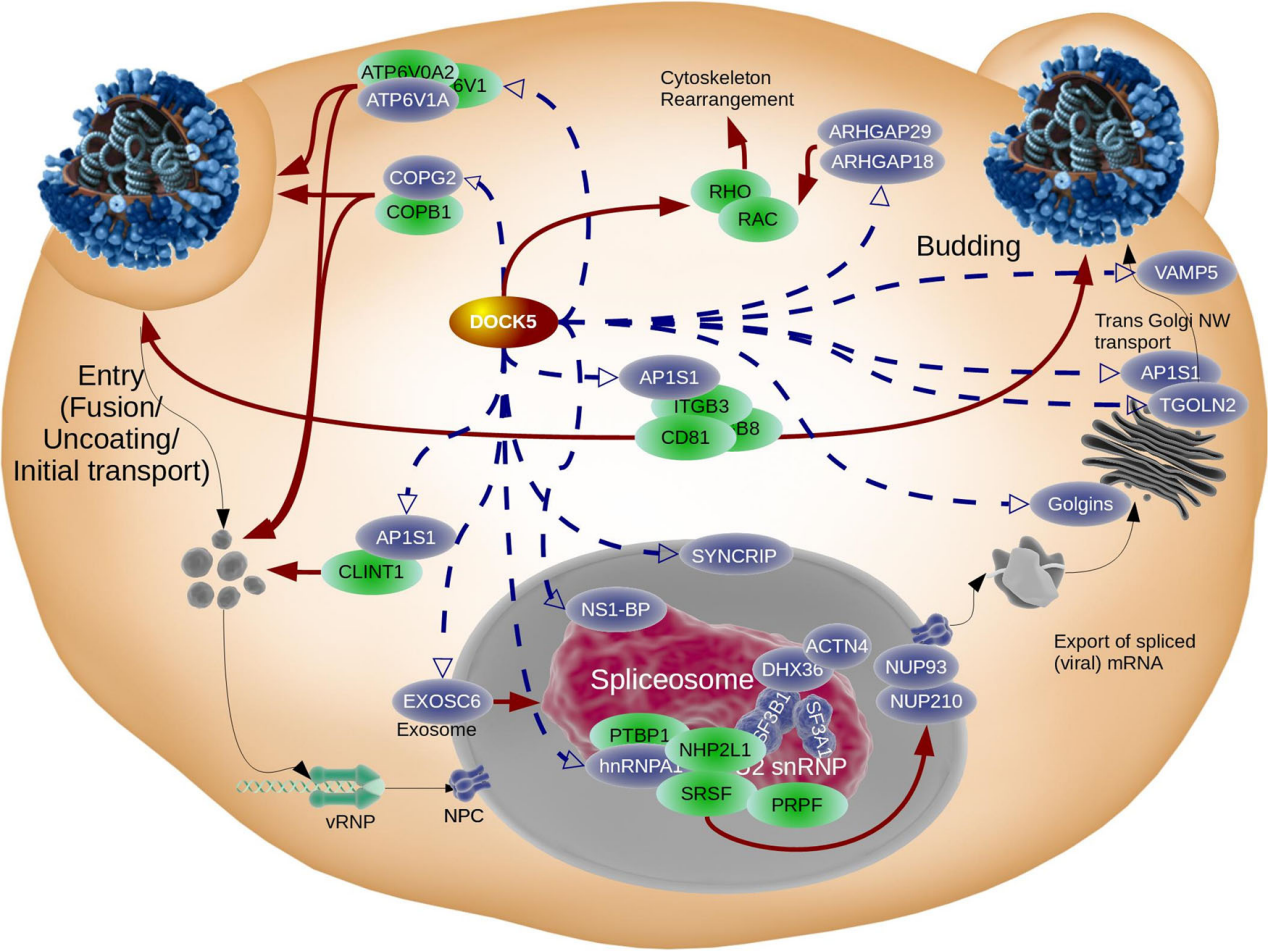
*DOCK5* is a key regulator for splicing and transport. Virus entry and initial transport to the nucleus is shown with regulatory links to the spliceosome, intracellular trafficking, and control of budding. In particular, *CD81* potentially controls both entry/initial transport as well as budding. *DOCK5* causally influences the V-type ATPases and *CLINT1* with respect to transport, as well as *NS1* *BP*, potentially for splicing. As a RAC specific GEF, *DOCK5* transduces signals for cytoskeleton rearrangement as a host defense response against IAV infection. *DOCK5* also down-regulates splicing by directly modulating *hnRNPA1*, *NS1* *BP*, and *SYNCRIP* as well as *SF3A1*/*SF3B1* and *DHX36*, in addition to down-regulation of *NUP93* and *NUP210*. Blue colored nodes are down-regulated by *DOCK5*. Green nodes are in the *DOCK5-*CCGS(7) neighborhood but not causally controlled by *DOCK5*. Closely spaced nodes indicate protein–protein interaction, black solid lines are material transport, blue dashed lines indicate *DOCK5* induced gene regulation, solid red lines denote induced functions based on published studies (see text)

In particular, we have identified three key processes in the centered network: (i) vesicle transport, (ii) pre-mRNA processing, and (iii) host defense. Figure 9 shows an overview of cellular processes, in particular vesicle trafficking and splicing, controlled by *DOCK5*. Cellular transport and viral trafficking are essential processes during the viral life cycle and the data suggest that *DOCK5* could modulate influenza virus trafficking within the cell. *DOCK5* also potentially influences gene regulation of trafficking-relevant genes. The trans-Golgi network protein 2 (*TGOLN2*), golgins, and the Golgi phosphoprotein 3 display highly conserved interactions between each other as well as with *DOCK5* (Fig. 9 rhs/”Budding”). Additional vesicle trafficking-associated genes, such as *VAMP5*, are highly up-regulated during IAV infection. *VAMP5* may function together with *TGOLN2* in the trans-Golgi complex (Fig. 9 rhs/”Budding”) though *VAMP5* does not mediate vesicle fusion with plasma membrane t-SNAREs.^54^ Loss of *DOCK5* significantly decreases *VAMP5* expression, indicating the importance of *DOCK5* for these cellular processes.

Other genes, such as a member of the adaptor protein complex 1 (*AP1S2*), which mediates the recruitment of clathrin to the Golgi complex, as well as the V-type ATPases, relevant for acidification and fusion of the cellular compartments, seem to be transcriptionally controlled by *DOCK5*. Together with *COPG2*, another cellular player for early processes during the influenza life cycle (i.e., uncoating and fusion, which is mediated by viral hemagglutinin, HA), as well as budding, *DOCK5* potentially modulates these first steps of IAV entry via *AP1S1*, *ATP6V1A*, and *COPG2* (Fig. 9 lhs/”Entry”). *DOCK5* may function in a regulatory feedback loop between COPI and *NS1* *BP*. Another potential regulatory dependency was observed between transcription factor *JUN* and *DOCK5*. According to LINCS data, *JUN* down-regulates *DOCK5*. Conversely, *JUN* expression is significantly reduced under *DOCK5-*ko conditions, indicating an (indirect) activation by *DOCK5* and the potential existence of a feedback loop.

The spliceosome, which is responsible for mRNA processing and *NS1BP* in particular, is another target that is most likely modulated by *DOCK5*. *NS1BP* binds to the predominantly cytosolic *SYNCRIP*, which itself is a member of the heterogeneous nuclear ribonucleoproteins. Other members of this complex, which are all controlled by *DOCK5*, are *hnRNFA1*, *hnRNPA3*, *hnRNPD*, *hnRNPR*, and *hnRNPU*, together with the splicing factor 3 components *SF3A1*, *SF3A2*, and *SF3B1* (Fig. 9 center/”Spliceosome”). *DOCK5* seems to be required for the proper function of the splicing machinery, by potentially mediating transport of splicing factors (via *KPNA4* and *TNPO1*), such as *hnRNPA1* between the cytoplasm and the nucleus, or by directly modulating alternative splicing responsible genes, such as *EXOSC6* and *DDX17*. Required influenza host factors, such as splicing genes (*PTBP1* and *SF3A1*, nuclear pore protein *NUP98)* as well as trafficking genes, are differentially spliced by *DOCK5* induction. Compared to the modulation of mRNA processing and splicing induced by NS1, *DOCK5* seems to be required for splicing of genes relevant for the influenza life cycle.

Although not the predominant function, *DOCK5* may be responsible for inhibiting specific host defense mechanisms to promote viral replication. We were able to identify 2 host defense processes that were up-regulated by *DOCK5*—the interferon-induced gene *ISG20* and cytokine *NAMPT*.

Availability of data from different cell lines and IAV strains, with different multiplicity of infection scenarios, allowed us to use a consensus approach at all levels of our multi-scale analysis, including capturing a consensus environment. This facilitated the discovery of universal processes that are essential during IAV infection. Thus, overall evidence indicates that *DOCK5* plays an important role in the gene-regulatory networks that potentially modulate host processes required for influenza infection by regulating intra-cellular trafficking and splicing, as well as subverting host defenses.

By combining an integrative network approach, a state-of-the-art gene knockout technique, and RNAseq data, this study uncovered and validated fundamental patterns of molecular responses, intrinsic structures of gene co-regulation, and novel key targets in influenza virus infection. Our findings pave a way for further functional investigations to identify novel therapeutic targets against influenza infection.

## Methods

A brief description of key methods and sample description is provided below, whereas complete details are discussed in the supplement.

### Modulation of virus growth and validation

To abolish the expression of functional DOCK5 proteins in A549 cells, the CRISPR/Cas9 genome editing system was used to introduce frameshift deletions into the *DOCK5* coding region of A549 cells. Briefly, CRISPR sgRNA was designed using the CRISPR Design tool^45^ and two pairs of oligo nucleotides (pair 1: 5′-CACCGTATGGCCCACGCGGACAATC-3′ and 5′-AAACGATTGTCCGCGTGGGCCATAC-3′; pair 2: 5′-CACCGGATAAATCGGAGCGAGCATT-3′ and 5′-AAACAATGCTCGCTCCGATTTATCC-3′) were selected, synthesized, individually annealed, and ligated into the pSpCas9(BB)-2A-Puro (PX459) V2.0 vector, following established protocols.^45^ The resultant plasmids pBZ321A6 and pBZ322A5 were transfected into human lung epithelial A549 cells. After puromycin selection, cells were cloned and sequenced to identify the ones with desired frameshift deletions. Four *DOCK5-*ko A549 clones (*DOCK5-*c20, c25, c28, and c41) were expanded by culture and compared to the wild type A549 (A549-WT) for their capacity to support the replication of influenza A virus strains.

To compare the replication kinetics of the influenza A virus in A549-wt and A549-*DOCK5-*ko cells, cells were seeded into 12-well plates to approximately 80% confluency (~2 × 10^5^ cells/well) at the time of infection. A/Puerto Rico/8/1934 (H1N1) and A/New York/238/2005 (H3N2) viruses were inoculated into the cells at MOI of 0.0005 (100 TCID_50_/well) and MOI of 0.005 (1000 TCID_50_/well), respectively. The supernatants were collected at days 1, 2, and 3 post-infection and titrated by TCID_50_ assay using MDCK cells. The inoculum was also back-titrated and the titer was used to represent the day 0 titer (Fig. 4). The medium for culture of the wild type and knockout A549 cells was F12K supplemented with 10% FBS and the medium for virus infection was MEM supplemented with 1% anti–anti (Thermo Fisher Scientific), 0.15% BSA fraction V (Thermo Fisher Scientific), and 1 μg/ml tosylsulfonyl phenylalanyl chloromethyl ketone (TPCK)-trypsin (Worthington, Lakewood, NJ). Three biological replicates were tested at each time point for each genotype, resulting in a total of 24 samples per each of the two viruses. A power analysis based on Cohen’s effect size estimate^55^ indicates that our experimental design will allow a ~80% power to detect an effect size of larger than 0.6 in viral production rate difference between *DOCK5* KO and wild-type.

A representative A549-*DOCK5-*ko clone, *DOCK5-*c28, was selected to determine the effects of DOCK5 knockout on global gene expression upon influenza virus infection. A549-WT and A549-*DOCK5-*c28 cells were each infected at MOI of 0.5 TCID_50_/cell of the PR8 (H1N1) virus, the A/New York/238/2015 (H3N2) virus, or Mock infected. Cells were harvested at 1 and 2 days post infection; total cellular RNA was extracted, and genomic DNA removed by DNase treatment. The frameshift deletion in the *DOCK5-*c28 clone compared to the *DOCK5-*wt is shown in Fig. S9.

### Analysis of RNA sequencing data

Single-ended RNA-seq data was generated using the Illumina HiSeq 2500 platform. The sequencing reads were aligned to the human hg19 genome using star aligner (version 2.5.0b). Following read alignment, featureCounts^56^ was used to quantify gene expression at the gene and exon levels based on Ensembl gene model GRCh37.70. Genes with at least 1 count per million (CPM) reads in at least one sample were considered expressed, otherwise absent and hence discarded. The gene level read counts data was normalized,^57^ multi-dimensional scaling, and cluster analysis were performed to check for potential sample outliers.

### Quantification of gene expression (RT-qPCR)

Wild type A549 and *DOCK5-*ko clones (c20, c25, c28, and c41) were infected as described above, and extracted RNA was used as a template for RT-qPCR. The RT-qPCR was performed using the TaqMan® RNA-to-Ct™ 1-Step Kit and the TaqMan® Gene Expression Assays (Thermo Fisher Scientific, Inc.). The assay IDs are Hs01103582_s1 for *JUN*, Hs01105383_g1 for *VAMP5*, Hs00158122_m1 for *ISG20*, Hs00353740 and Hs99999905_m1 for *NR3C1*, Hs00227848_m1 for *DOCK5*, and Hs00213893_m1 for *WBSCR22*. *GAPDH* was used as an internal control for the quantification because its level is unchanged upon virus infection. The 2^−ΔΔCT^ method was used to analyze the relative changes in gene expression.^58^

### Data sets and sample processing

We compiled from GEO 8 microarray expression profiles (Table 1) of human cell cultures infected with different IAV subtypes and strains yielding 12 distinct data sets. We subjected the expression data to log2 transformation and quantile normalization.

### Identification of differentially expressed genes

We used three distinct methods to identify differentially expressed genes. The *t*-test was used to identify differentially expressed genes (DEGs) between case and control. We used one-way ANOVA to determine significantly responding genes (SRGs) depending on time post infection as a single independent factor. Multi-factorial analysis of temporal expression data between wildtype and *DOCK5-*ko genotypes were performed by a hierarchical linear model (hLM) after Limma.^59^ Significantly up-regulated and down-regulated genes depending on time post infection were identified by the non-parametric Jonckheere trend analysis.^25^

### Gene co-expression network analysis

We performed weighted gene co-expression network analysis (WGCNA) to identify 1191 modules of highly co-expressed genes from the assembled 12 data sets. Using the Jaccard-Needham dissimilarity measure for assessing module–module similarity and the hierarchical clustering analysis, the 1191 modules were further grouped into 282 clusters, i.e., consensus modules.

### Co-expression modules and consensus modules (CMs)

We performed WGCNA^17,18^ to identify 1191 modules of highly co-regulated genes from the assembled 12 data sets. We further identified consensus modules conserved across multiple data sets based on the hierarchical clustering analysis of using the Jaccard-Needham dissimilarity matrix for the 1191 modules.

The total relevance of each consensus module to influenza infection was calculated by summarizing the enrichment of the DEG signatures: $G_j = \mathop {\prod }\nolimits_i g_{ji}$, where, $g_{ji}$ is the relevance of a consensus *j* to a signature *i*. $g_{ji}$ is defined as $\left( {{\rm max}_j\left( {r_{ji}} \right) + 1 - r_{ji}} \right)/\mathop {\sum }\nolimits_j r_{ji}$, where $r_{ji}$ is the ranking order of the significance level of the overlap between the consensus module *j* and the signature *i*.

### Enrichment analysis and internal verification of data

To functionally annotate gene signatures and gene modules identified in this study, we performed enrichment analysis of the established pathways and signatures including the gene ontology (GO) categories and MSigDB, and the subject area specific gene sets including influenza host factors, inflammasome, interferome, and InnateDB.

### Data availability statement

Analytic results are available in a large number of supplementary tables. All raw RNA-sequencing data (single FASTQ files) as well as the processed CPM matrix from this study have been deposited into the Gene Expression Omnibus (GEO) under Accession Number GSE104168.

### Code availability

The R code and the R package WINA for the coexpression network analysis are available at doi:10.7303/syn7221264.2.

## Electronic supplementary material

Supplementary Tables

Supplementary Methods and Figures
