# Supplementary material for: Integrative gene network analysis identifies key signatures, intrinsic networks and host factors for influenza virus A infections
Source: NPJ Syst Biol Appl. 2017 Dec 4;3:35. doi: 10.1038/s41540-017-0036-x (PMC5712526; doi:10.1038/s41540-017-0036-x)
Supplement: Supplementary file 2 — Supplementary Methods and Figures [file 41540_2017_36_MOESM2_ESM.docx]

# Integrative Gene Network Analysis Identifies Key Signatures, Intrinsic Networks and Essential Host Factors for Influenza Virus A Infections

## Supplementary Information

## Supplementary Methods

**Analysis of RNA Sequencing Data**

Single-ended RNA-seq data was generated using the Illumina HiSeq 2500 platform. The sequencing reads were aligned to the human hg19 genome using star aligner (version 2.5.0b). Following read alignment, featureCounts [[1](#_ENREF_1)] was used to quantify the gene expression at the gene and exon level based on Ensembl gene model GRCh37.70. Genes with at least 1 count per million (CPM) reads in at least 1 sample were considered expressed, otherwise absent and hence discarded. Next, the gene level read counts data was normalized as CPM using the trimmed mean of M-values normalization (TMM) method [[2](#_ENREF_2)] to adjust for sequencing library size difference. Multi-dimensional scaling (MDS) and cluster analysis were performed using the R programming language to check for potential sample outliers. For differential exon usage analysis, exons with at least 1 CPM in at least 1 sample were selected and normalized by the TMM method.

### Data Pre-processing

- 1. Microarray gene expression profiles were directly accessed from GEO and processed via R/Bioconductor. Relative expression fold-changes against the control (time point zero, i.e., mock-infection or time-point matched mock-infections in the case of time-dependent mock-infection measurements) were calculated. Individual datasets were log2 transformed and quantile normalized. For differential expression analysis, all time series data from all samples were aligned
  2. Further pre-processing steps included the filtering of unresponsive genes/probes. For this purpose, the coefficient of variation was calculated and probes only accepted that were in the top 86% of the corresponding cumulative distribution. In addition to the highly responsive probes, probes of known influenza response genes as well as probes of DEGs (with 5% FDR) were also included. We subjected the time series data to correlation and hierarchical cluster analysis.

### Differential Expression, Time-series Trend Analysis

Differential gene expression as well as differential splicing of exons between different genotypes/treatments were predicted by hierarchical linear model (hLM) analysis using the Bioconductor package limma [[3](#_ENREF_3)]. To adjust for multiple tests, the false discovery rate (FDR) of the differential expression test was estimated using the Benjamini–Hochberg (BH) method [[4](#_ENREF_4)]. Genes or exons with FDR less than 0.05 and absolute log_2_ fold change larger than log_2_(1.2) were considered significant. Additional fold change cutoffs of log_2_(1.5), log_2_(2), and log_2_(4) were considered in the case of differential expression.

“Significantly Responding Genes (probes)” across the measured time series data were identified to evaluate the significance of expressed genes. For this purpose an analysis of variance (ANOVA) test was used. The one-way ANOVA is a parametric method for testing if samples originate from the same distribution. Calculated p-values after ANOVA testing were corrected against false discoveries by the Benjamini-Hochberg correction, as well as by direct estimation and random sampling.

The up- and downward trend for each expressed gene was also determined to identify differentially expressed genes across time series. For this purpose, Jonckheere’s trend test was used to determine an *a priori* ordering of the medians of the gene expression replicates in time.

### Gene co-expression network

Weighted gene co-expression network analysis (WGCNA) [[5](#_ENREF_5)] was performed to identify the gene modules with coordinated expression patterns for each brain region. Briefly, Pearson’s correlation coefficients were calculated between all pairs of probes. Next, the correlation matrix was converted into an adjacency matrix using a power function *f*(*x*) = *x^β^*, where *x* was the element of the correlation matrix and parameter *β* was determined such that the resulting adjacency matrix was approximately scale-free [[5](#_ENREF_5)]. The adjacency matrix was subsequently transformed into a topological overlap matrix (TOM) [[6](#_ENREF_6)], which captured both the direct and indirect interactions between a pair of probes. Average linkage hierarchical clustering was then employed to cluster probes based on the TOM. Finally a tree cutting algorithm [[7](#_ENREF_7)] was used to dynamically cut the hierarchical clustering dendrogram branches into highly connected modules, each of which was assigned a distinct color code.

For further identification of consensus modules, we used the Jaccard-Needham dissimilarity measure to determine the similarity between any 2 co-expression modules from all the datasets and then employed the hierarchical clustering analysis to identify clusters of similar modules, i.e., consensus modules.

### Gene Set Enrichment Analysis

Simple, non-weighted gene set enrichment analysis was performed by using a variety of different gene set databases, including GO [[8](#_ENREF_8)], MSigDB [[9](#_ENREF_9)], influenza host factors [[10](#_ENREF_10), [11](#_ENREF_11)], the inflammasome [[12](#_ENREF_12)], the Interferon Stimulated Genes (ISGs) [[13](#_ENREF_13)], the known host defense factors from InnateDB [[14](#_ENREF_14)], and transcription factors from the ENCODE project [[15](#_ENREF_15)]. As standard procedure the Fisher Exact Test was employed using Bonferroni correction for multiple testing.

**Details on the Validation of the *DOCK5*-centered network**

Validation analysis was done for the DEG signatures from 15 configurations (**Fig. S5** and **S6**; **Table S26**). Among 282 consensus modules, 5 are significantly enriched for the genes up- or down-regulated by *DOCK5* during MOCK infection, 27 for the genes upregulated by *DOCK5* knockout during H1N1 infection, 31 for the genes upregulated by *DOCK5* knockout during H3N2 infection, 7 for the genes down-regulated by *DOCK5* knockout during H1N1 infection, and 16 for the genes down-regulated by *DOCK5* knockout during H3N2 infection. Eleven of the top 13 modules in **Table 3** are significantly enriched for sgDOCK5-DEGS. For example, the 1^st^, 3^rd^, 4^th^ and 5^th^ modules are enriched for the DOCK5-ko signatures with FET p = 9.86e-28 (2.2-fold), 5.98e-12 (2.2-fold), 1.04e-12 (1.6-fold) and 5.79e-13 (1.8-fold), respectively. These modules have different functions such as viral reproduction, single-organism cellular process, and organelle organization, indicating that DOCK5 regulates a diversity of biological processes during IAV infection and thus is an essential target of IAV infection.

Regarding the *DOCK5* centered network, DOCK5-CCGS(7) shares 786 genes with sgDOCK5-DEG^+^ (FET p = 8.32e-68, 1.75-fold; **Figure 6A**). This intersection is involved in cell-cell adhesion and carbohydrate metabolic processes, in particular involving potentially vesicle-inducing glycosyltransferases [[16](#_ENREF_16)] (**Table S37**). Significant enrichment of the inflammasome signature in DOCK5-CCGS(7) (FET p=4.3e-5, 1.2-fold change), and sgDOCK5-DEG^+^ (FET p=3.1e-5, 1.2-fold change), as well as their intersection, were also observed (FET p=5.9e-6, 1.5-fold change – **Table S38**). Moreover, 73 of the 786 genes are in InnateDB (FET P=0.04, 1.4-fold change), indicating a repression of innate immune system functions by *DOCK5*. Among the 73 genes common to DOCK5-CCGS(7), sgDOCK5-DEG^+^ and InnateDB, 4 (*ADAR*, *FGFR1*, *IL6* and *STAT1*) are related to the innate immune response according to InnateDB and they are up-regulated in 6 or more datasets, 29 are significantly down-regulated in the majority of the 12 datasets including suppressor of cytokine signaling 6 (*SOCS6*) and SMAD family member 3 (*SMAD3*) [[17](#_ENREF_17)]. Both *SOCS6* and *SMAD3* are highly responsive to IAV infection and are not only differentially expressed in 10/12 datasets but also down-regulated in half of the datasets.

Highly significant overlap among DOCK5-CCGS(7), SRGs(7), JTGs(7) and sgDOCK5-DEG^+^ by the Super Exact Test (SET) [[18](#_ENREF_18)] strongly validated our predicted *DOCK5-*centered network, as shown in **Figure 7**. sgDOCK5-DEG^+^ highly significantly overlaps with (i) the genes common to DOCK5-CCGS(7), SRGs(7) and JTGs^down^(7) (SET P<1e-320, 86-fold), (ii) those common to both DOCK5-CCGS(7) and SRGs(7) (SET P=2.2e-249,10.2-fold) and (iii) those common to both SRGs(7) and JTGs^down^(7) (SET P= 7.7e-299,17.9-fold). There is a relatively weaker but nevertheless significant overlap between sgDOCK5-DEG^+^ and the upregulated genes during influenza infection, JTGs^up^(7). For example, the overlap among the four sets including sgDOCK5-DEG^+^, DOCK5-CCGS(7), SRGs(7) and JTGs^up^(7) has a SET P value of 3.4e-15 (20-fold).

Among the genes shared by JTGs(7), DOCK5-CCGS(7) and sgDOCK5-DEG^+^, 331 have a significant down-regulation trend in at least 7 datasets (SET p=2.8e-219, 9.2-fold change) and 25 have a significant up-regulation trend (SET P=2.3e-5, 2.6-fold), including host defense genes such as *IL6*, *PSME1*, and *STAT1*. As shown in **Figure S5**, the intersection among DOCK5-CCGS(7), SRGs(7) and sgDOCK5-DEG^+^ is enriched for the adherence junction pathway (FET p=0.05, 5.0-fold change). Whereas, 435 genes shared by only DOCK5-CCGS(7) and sgDOCK5-DEG^+^ are enriched for carbohydrate metabolism via glycosyltransferases (FET p=1.8e-4, 2.3-fold change), vesicle-mediated transport (FET p=0.033, 1.9-fold change) and mRNA processing (FET p=0.019, 7.8-fold change). These findings confirm the potential role of *DOCK5* in modulating cell-adhesion/cytokinesis, vesicle-mediated transport and immune system processes.

The intersection of DOCK5-CCGS(7), SRGs(7) and sgDOCK5-DEG^+^ includes 24 interferon stimulated genes, i.e. ISGs, (FET p=1.6e-5, 3.2-fold change; *ABHD2*, *AKAP13*, *BBX*, *CD44*, *CD47*, *FANCI*, *GTF2I*, *IL6*, *ISG20*, *NAMPT*, *NFIB*, *NS1BP*, *RIF1*, *RPA1*, *RRP1B*, *RXRA*, *SCAMP1*, *SCD*, *SCD5*, *SERINC5*, *SIRT3*, *STAT1*, *TBL1XR1*, and *WWTR1* – **Table S29**). Of these 24 genes, only *ISG20*, *IL6* and *STAT1* are predominantly up-regulated across the datasets while all other genes are either up- or down-regulated in comparable numbers of datasets. *ISG20* shows a greater difference between the *DOCK5*-wt cells and *DOCK5*-ko cells. This particular interferon-stimulated gene functions as an antiviral ribonuclease, potentially degrading viral RNA or indirectly affecting cellular factors required for viral replication [[19](#_ENREF_19)]. In the *DOCK5*-wt cells, *ISG20* was up-regulated by over 8 fold 2 days post-infection (H1N1: 9.6-fold change; H3N2: 8.3-fold change). In the *DOCK5*-ko cells, the expression of *ISG20* increased more dramatically after 2 days after post-infection (H1N1: 20.3-fold change; H3N2: 27.5-fold change). *ABHD2*, *NS1BP* and *RPA1* were down-regulated in 11 out of the 12 datasets. The expression level of *ISG20* was further quantified by qPCR.

Genes in the overlap between DOCK5-CCGS(7) and sgDOCK5-DEG^-^ include the vesicle associated membrane protein 5 (*VAMP5*), cytokines *CCL5*, *CXCL11*, *IRF7*, *HBEGF*, *RRAD*, *MYLIP*, *JUN*, *CRY1*, *RGS16*, *FGFR1*, and *C8orf46*. *VAMP5* is significantly upregulated in *DOCK5*-wt cells 2 days post-infection (H1N1: 5.1-fold change; H3N2: 13.0-fold change). Under *DOCK5*-ko condition, upregulation of *VAMP5* is significantly reduced to 1.4-fold change (H1N1) and 1.7-fold change (H3N2) 2 days post-infection. According to the RNAseq experiments, the transcription factor and potential *DOCK5* regulator *JUN* itself is up-regulated in *DOCK5*-wt (H1N1: 2.1-fold change; H3N2: 2.2-fold change) and not in the DOCK5-ko cells (H1N1: 1.3-fold change; H3N2: no fold change) 2 days post-infection. In data from the Library of Integrated Network-based Cellular Signatures (LINCS), *DOCK5* is up-regulated by *JUN* knock-down (P=0.067, 1.2-fold change), indicating a modulation (suppression) of *DOCK5* transcription by *JUN*. The expression levels of both *JUN* and *VAMP5* were further quantified by qPCR.

### Rank-order of Modules

The relevance of each consensus module was accessed using enrichment for DEG signatures across time series from individual studies. These measurements were then summarized to rank order the consensus modules by computing a total relevance score $G_{j}=\prod_{i} g_{ji}$, where, $g_{ji}$ is the relevance of a module ***j*** to a signature ***i***. $g_{ji}$ is defined as ${({max}_{j}(r_{ji})+1-r_{ji})}/{\sum_{j} r_{ji}}$, where $r_{ji}$ is the ranking order of the enrichment statistics for the consensus module t ***j*** and the signature ***i***.

## Supplementary References

1. Liao, Y., G.K. Smyth, and W. Shi, *featureCounts: an efficient general purpose program for assigning sequence reads to genomic features.* Bioinformatics, 2014. **30**(7): p. 923-930.

2. Robinson, M.D., D.J. McCarthy, and G.K. Smyth, *edgeR: a Bioconductor package for differential expression analysis of digital gene expression data.* Bioinformatics, 2010. **26**(1): p. 139-140.

3. Ritchie, M.E., et al., *limma powers differential expression analyses for RNA-sequencing and microarray studies.* Nucleic Acids Research, 2015. **43**(7): p. e47.

4. Benjamini, Y. and Y. Hochberg, *Controlling the False Discovery Rate: A Practical and Powerful Approach to Multiple Testing.* Journal of the Royal Statistical Society. Series B (Methodological), 1995. **57**(1): p. 289-300.

5. Zhang, B. and S. Horvath, *A General Framework for Weighted Gene Co-Expression Network Analysis.* Statistical Applications in Genetics and Molecular Biology, 2005. **4**(1): p. Article 17.

6. Ravasz, E., et al., *Hierarchical Organization of Modularity in Metabolic Networks.* Science, 2002. **297**(5586): p. 1551-1555.

7. Langfelder, P., B. Zhang, and S. Horvath, *Defining clusters from a hierarchical cluster tree: the Dynamic Tree Cut package for R.* Bioinformatics, 2008. **24**(5): p. 719-720.

8. Gene Ontology, C., *Gene Ontology Consortium: going forward.* Nucleic Acids Res, 2015. **43**(Database issue): p. D1049-56.

9. Subramanian, A., et al., *Gene set enrichment analysis: a knowledge-based approach for interpreting genome-wide expression profiles.* Proc Natl Acad Sci U S A, 2005. **102**(43): p. 15545-50.

10. Watanabe, T., S. Watanabe, and Y. Kawaoka, *Cellular networks involved in the influenza virus life cycle.* Cell Host Microbe, 2010. **7**(6): p. 427-39.

11. Ward, S.E., et al., *Host modulators of H1N1 cytopathogenicity.* PLoS One, 2012. **7**(8): p. e39284.

12. Wang, I.M., et al., *Systems analysis of eleven rodent disease models reveals an inflammatome signature and key drivers.* Mol Syst Biol, 2012. **8**: p. 594.

13. Rusinova, I., et al., *Interferome v2.0: an updated database of annotated interferon-regulated genes.* Nucleic Acids Res, 2013. **41**(Database issue): p. D1040-6.

14. Breuer, K., et al., *InnateDB: systems biology of innate immunity and beyond--recent updates and continuing curation.* Nucleic Acids Res, 2013. **41**(Database issue): p. D1228-33.

15. Consortium, E.P., *An integrated encyclopedia of DNA elements in the human genome.* Nature, 2012. **489**(7414): p. 57-74.

16. Guzman-Aranguez, A., et al., *Targeted disruption of core 1 beta1,3-galactosyltransferase (C1galt1) induces apical endocytic trafficking in human corneal keratinocytes.* PLoS One, 2012. **7**(5): p. e36628.

17. Roberts, A.B., et al., *Smad3: A Key Player in Pathogenetic Mechanisms Dependent on TGF‐β.* Annals of the New York Academy of Sciences, 2003. **995**(1): p. 1-10.

18. Wang, M., Y. Zhao, and B. Zhang, *Efficient Test and Visualization of Multi-Set Intersections.* Sci Rep, 2015. **5**: p. 16923.

19. Espert, L., et al., *ISG20, a new interferon-induced RNase specific for single-stranded RNA, defines an alternative antiviral pathway against RNA genomic viruses.* Journal of Biological Chemistry, 2003. **278**(18): p. 16151-16158.

Supplementary Tables 1-38: Included in the spread sheet


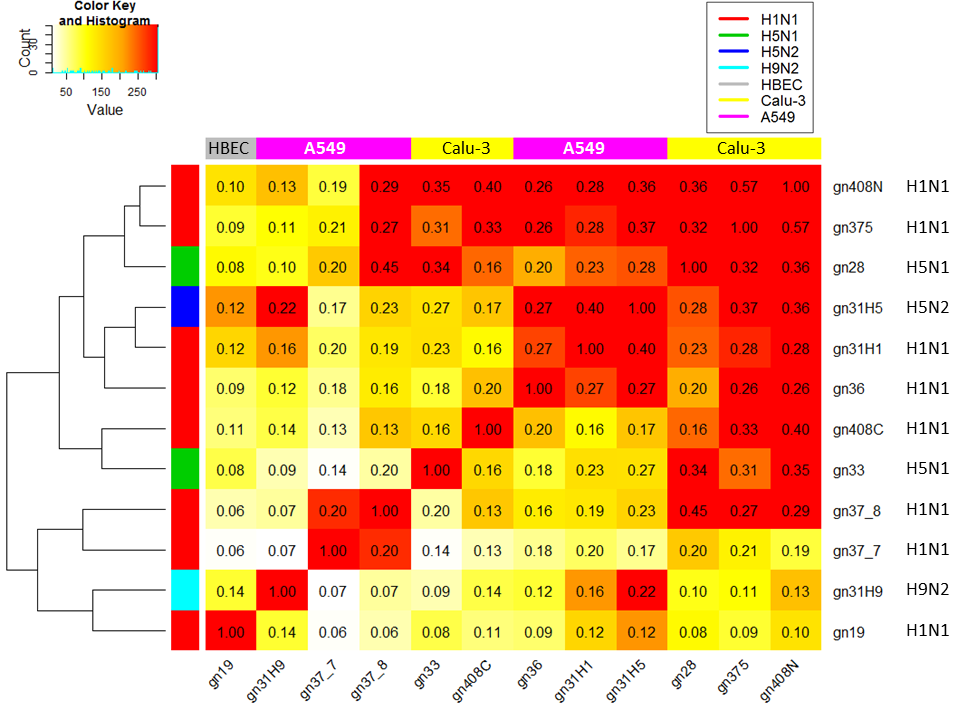


Supplementary Fig. 1: FET overlap between SRGs of individual gene expression datasets. The heat map shows the –log10(P-value) of the FET overlap, the numbers denote the fraction of the overlap compared to the maximum of the size of the individual datasets. Vertical side colors on the lhs refer to the virus strains, horizontal side colors at the top of the heatmap show the cell types. The FET P-values range between 1.37e-11 (white) and 0 (red).


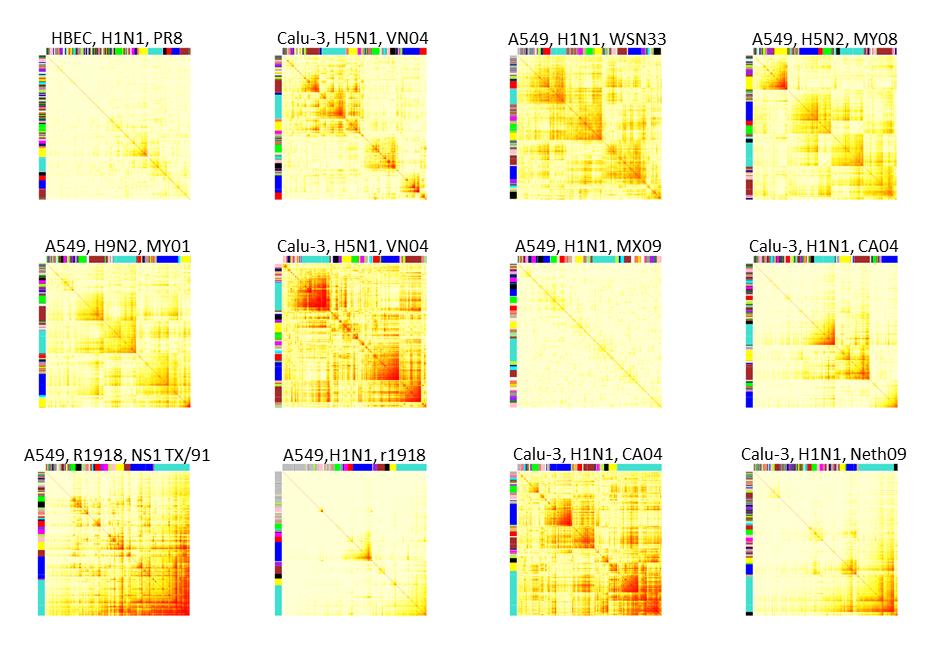


Supplementary Fig. 2: Weighted gene co-expression network analysis of 12 datasets assembled in this study. Gene expression data originate from cell-based studies on HBEC primary cells, as well as A549 and Calu-3 cell lines infected with different IAV subtypes and strains. These weighed co-expression networks include 1,191 modules. Each coexpression network is represented by a symmetric heat map in which rows and columns are genes and the red color intensity indicates the network connection strength between any pair of nodes (genes). The network modules highlighted as colored bars along the rows and columns were identified via an average linkage hierarchical clustering algorithm using topological overlap as the similarity metric.


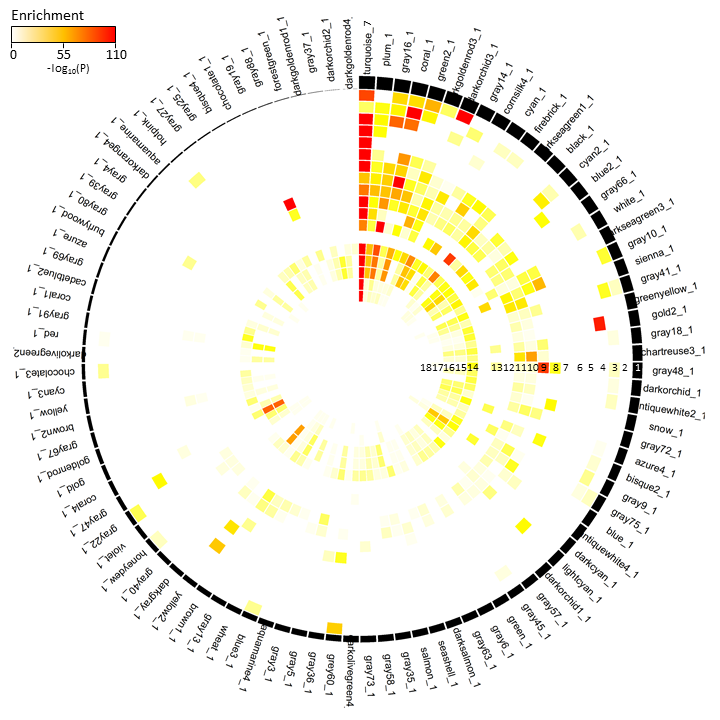


1. Rank

2. – 13. Individual SRG enrichment

14. Consensus DEG enrichment

15. Enrichment of consensus up-regulated genes JTGs^up^(7)

16. Enrichment of consensus down-regulated genes JTGs^down^(7)

17, 18. Enrichment of influenza infection targets (Watanabe influenza, Ward *et al*., resp.)

**Supplementary Fig. 3: A circular representation of the enrichment of the 100 best ranked consensus modules in various informative gene signatures or sets derived from this study and the literature.** The outmost track shows the consensus modules. From outside to inside, the bar chart in the first track represents the relative relevance score, the heat maps in the tracks 2–13 show the enrichment for individual SRG signatures, the heat map in the track 14 represents the enrichment for the consensus DEG signature, SRGs(7), the heat map in the track 15 shows the enrichment for the consensus up-regulated genes JTGs^up^(7), the heat map in the track 16 show the enrichment for the consensus down-regulated genes JTGs^down^(7), the heat map in the track 17) shows the enrichment for the Watanabe influenza targets, and the heat map in the track 18 shows the enrichment of the Ward et al. siRNA targets.


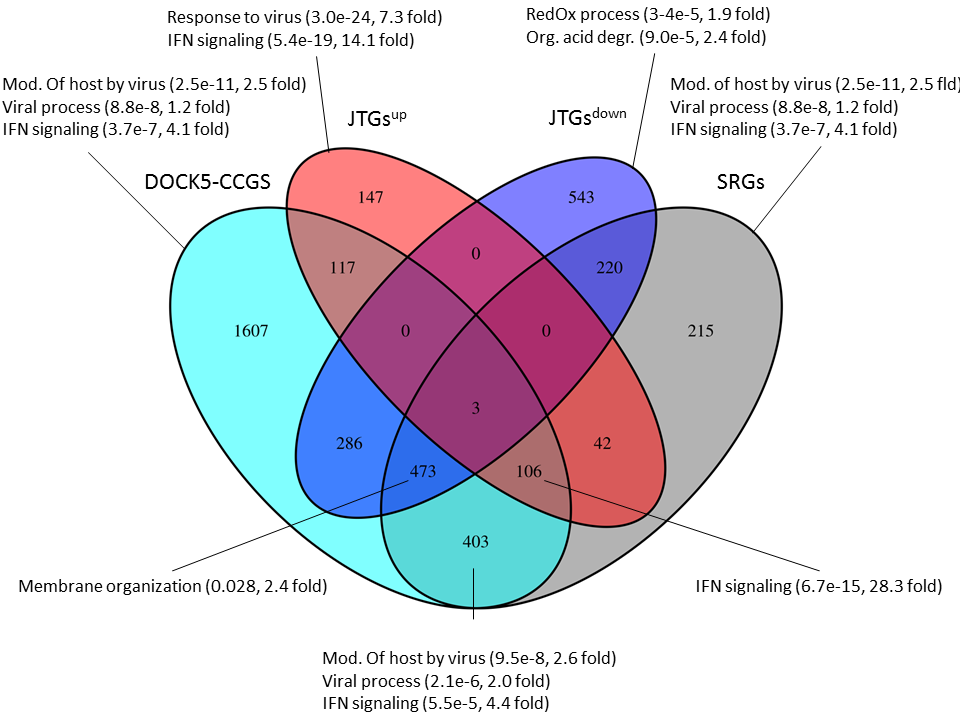


Supplementary Fig. 4: Venn diagram of the DOCK5-CCGS(7), SRGs(7), JTGs^up^(7), JTGs^down^(7) sets. The Venn diagram shows unique and conserved genes and their functions between the *DOCK5-*correlated consensus gene set (DOCK5-CCGS(7)), SRG(7), up- and down-regulated genes (JTGs^up^(7), JTGs^down^(7)). Numbers in parenthesis next to the biological functions denote the corrected P-value and the fold enrichment after FET.

**
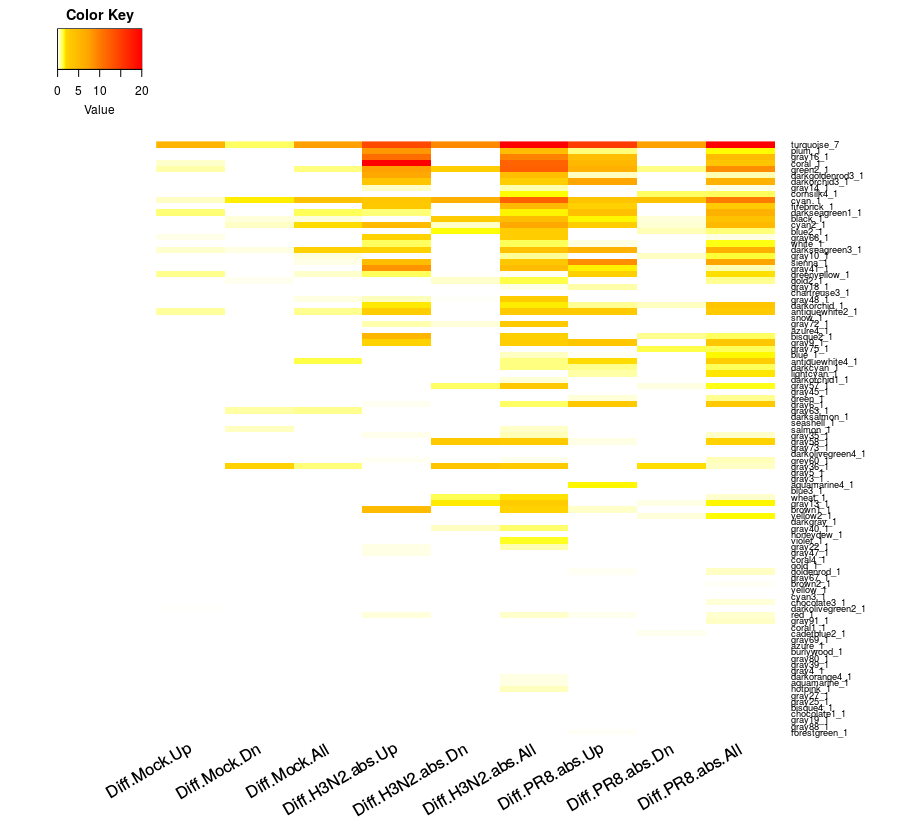
Supplementary Figure 5:** Enrichment of consensus modules (CMs) by sgDOCK5-DEGS. The best 100 CMs together with 9 of the 15 sgDOCK5-DEGS are shown. The remaining 6 sgDOCK5-DEGS do not show any significant enrichment. Modules are ranked from top to bottom by DEGs enrichment from individual expression profiles.**
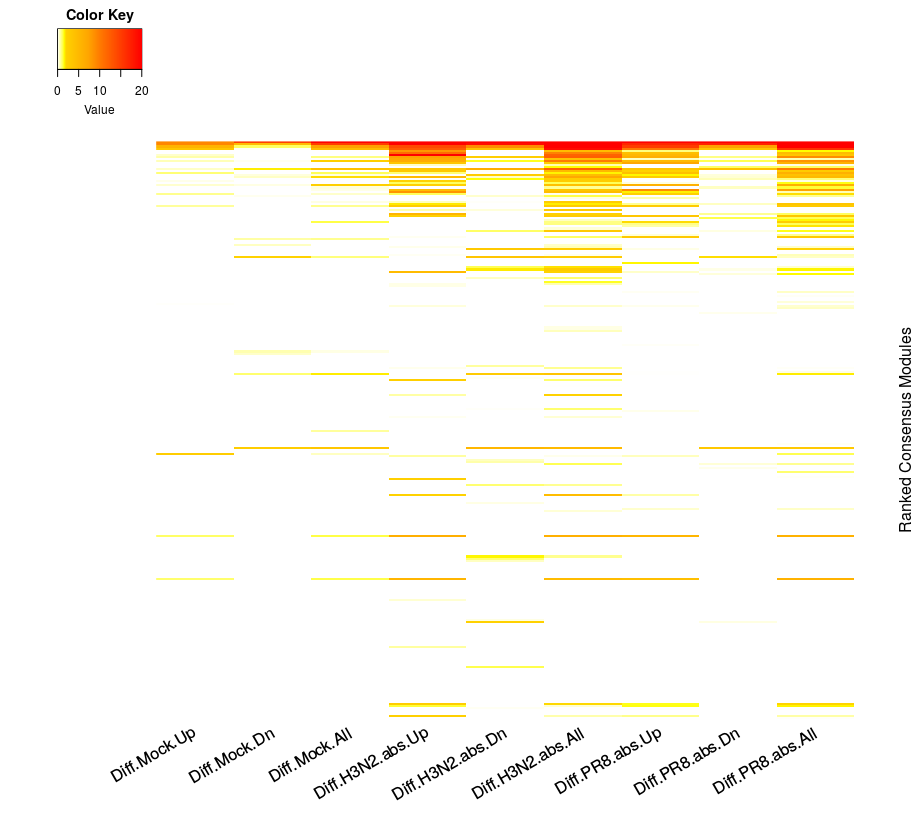
**

Supplementary Figure 6: Enrichment of sgDOCK5-DEGs in the consensus modules (CMs). All 282 CMs are shown together with 9 of the 15 sgDOCK5-DEGs. The minimal corrected p-value is 3.1e-83.


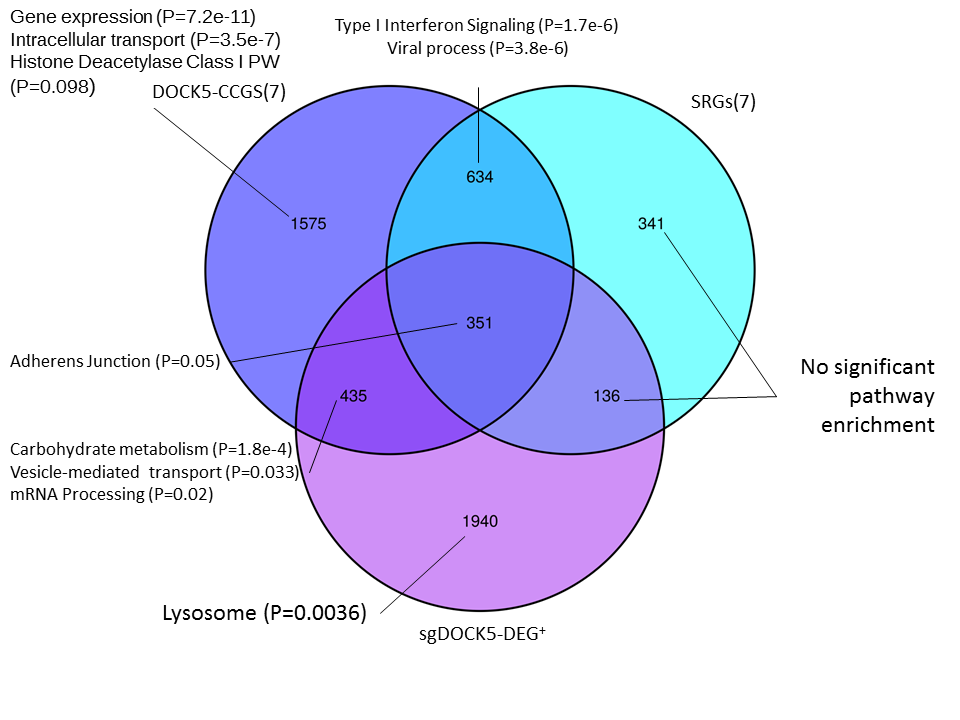
Supplementary Figure 7: Venn diagram of the *DOCK5*-correlated consensus gene set (DOCK5-CCGS(7)), ANOVA consensus 7 DEGs (SRGs(7)) and the genes repressed by *DOCK5* (sgDOCK5-DEG^+^).


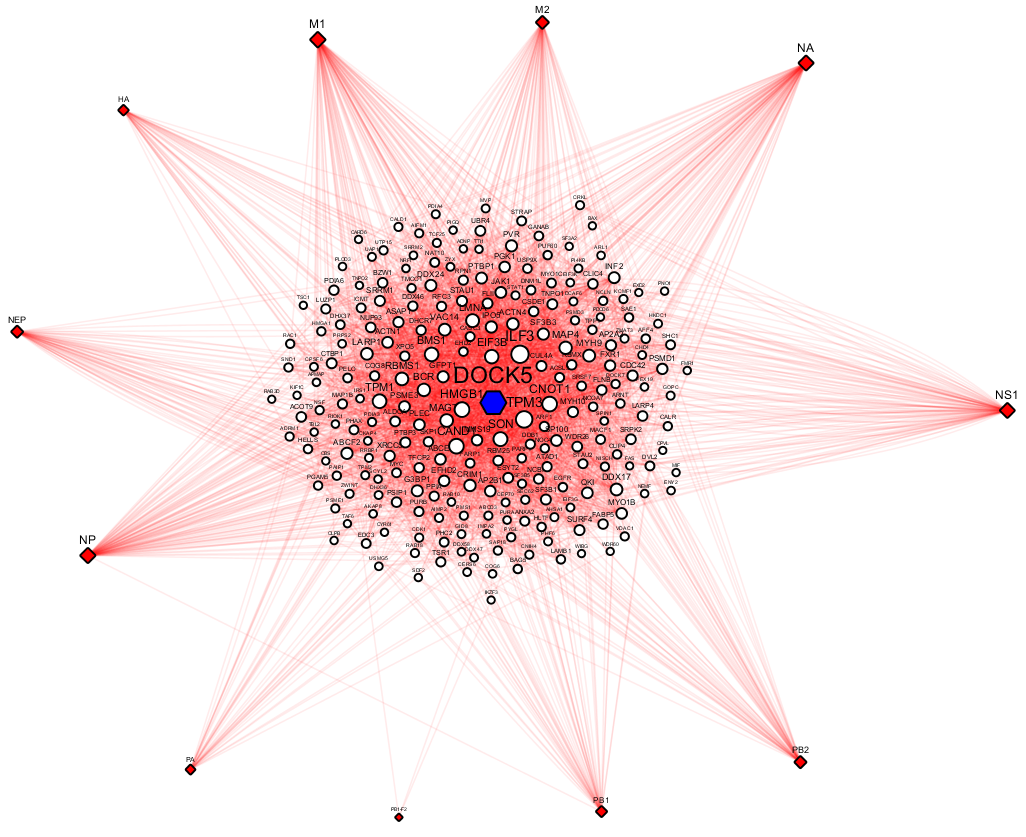


**Supplementary Figure 8:** Merged network between influenza host-factor protein-protein interaction network and DOCK5-CCGS(7). A network of 231 common host-factors (white nodes) that connect 11 influenza proteins (red diamonds) with DOCK5 (blue hexagon) is shown.


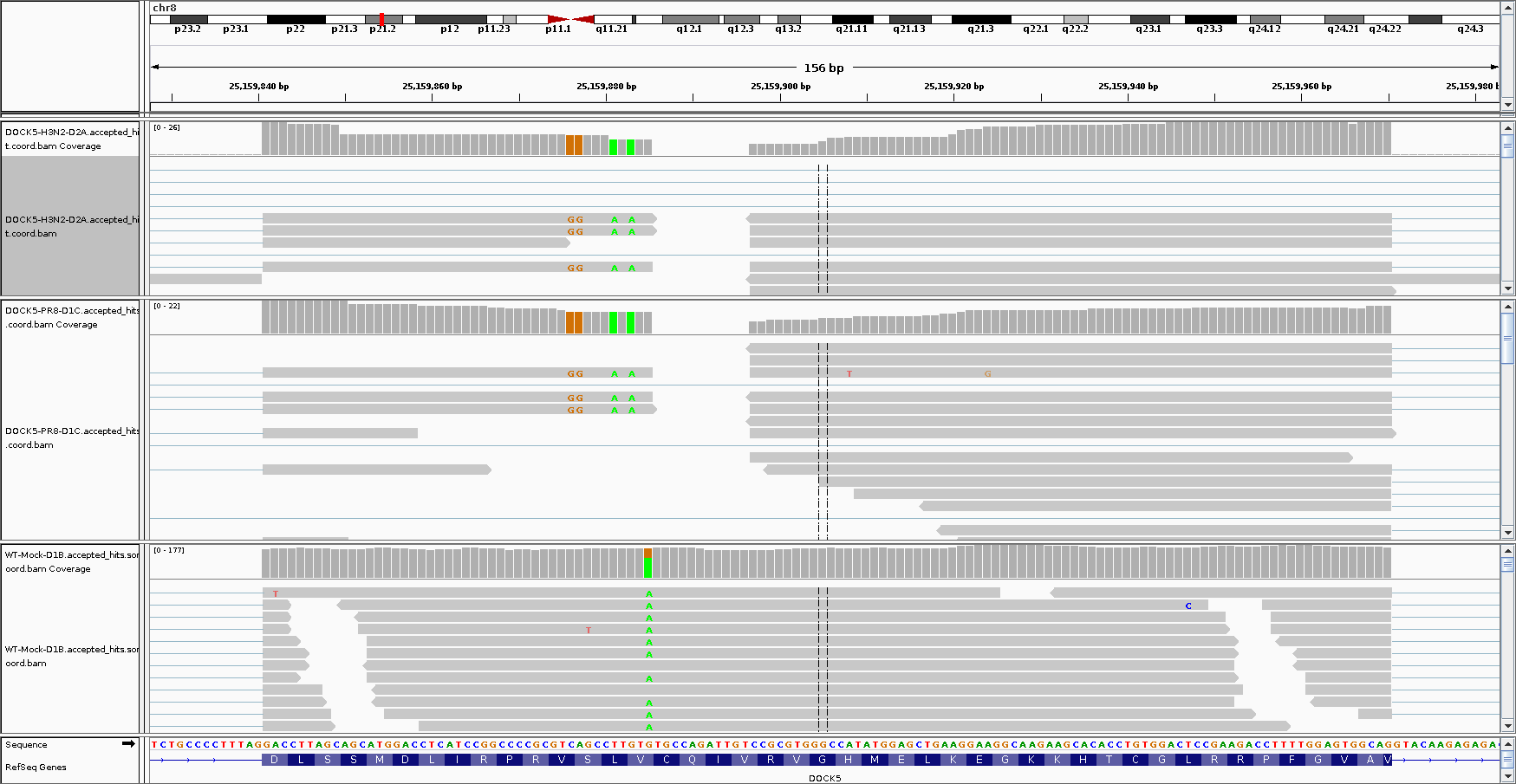


Supplementary Figure 9: Section of the frame-shift mutation in the *DOCK5*-ko cell line. A section of the *DOCK5*-ko, *DOCK5*-wt and reference genome is shown in the IGV genome browser.
